# A uniquely efficacious type of CFTR corrector with complementary mode of action

**DOI:** 10.1126/sciadv.adk1814

**Published:** 2024-03-01

**Authors:** Valentina Marchesin, Lucile Monnier, Peter Blattmann, Florent Chevillard, Christine Kuntz, Camille Forny, Judith Kamper, Rolf Studer, Alexandre Bossu, Eric A. Ertel, Oliver Nayler, Christine Brotschi, Jodi T. Williams, John Gatfield

**Affiliations:** Idorsia Pharmaceuticals Ltd., 4123 Allschwil, Switzerland.

## Abstract

Three distinct pharmacological corrector types (I, II, III) with different binding sites and additive behavior only partially rescue the F508del-cystic fibrosis transmembrane conductance regulator (CFTR) folding and trafficking defect observed in cystic fibrosis. We describe uniquely effective, macrocyclic CFTR correctors that were additive to the known corrector types, exerting a complementary “type IV” corrector mechanism. Macrocycles achieved wild-type–like folding efficiency of F508del-CFTR at the endoplasmic reticulum and normalized CFTR currents in reconstituted patient-derived bronchial epithelium. Using photo-activatable macrocycles, docking studies and site-directed mutagenesis a highly probable binding site and pose for type IV correctors was identified in a cavity between lasso helix-1 (Lh1) and transmembrane helix-1 of membrane spanning domain (MSD)-1, distinct from the known corrector binding sites. Since only F508del-CFTR fragments spanning from Lh1 until MSD2 responded to type IV correctors, these likely promote cotranslational assembly of Lh1, MSD1, and MSD2. Previously corrector-resistant CFTR folding mutants were also robustly rescued, suggesting substantial therapeutic potential for type IV correctors.

## Teaser

A CFTR corrector type with distinct binding site, complementary mode of action, and superior trafficking efficacy is described.

## INTRODUCTION

Cystic fibrosis is a common life-threatening genetic disorder caused by mutations in the cystic fibrosis transmembrane conductance regulator (*CFTR*) gene (*1*). CFTR is a chloride and bicarbonate channel expressed on the surface of secretory epithelia. Its absence or dysfunction compromises mucus hydration and fluidity leading to mucus accumulation and chronic inflammation in multiple organs and their progressive functional impairment, with the most severe, ultimately lethal effects in the lung (*2*, *3*). In recent decades, early diagnosis and symptomatic treatments have improved CF patients’ quality of life and extended their life expectancy (*4*). In the past 10 years, CFTR-targeted therapies (CFTR modulators) have further improved symptoms and quality of life, demonstrating that addressing the molecular defect in CF is an effective strategy and warrants further research (*3*).

CFTR is a 1480–amino acid transmembrane protein of the ABC transporter family. It consists of two membrane spanning domains (MSD1 and MSD2), two nucleotide binding domains (NBD1 and NBD2), and a regulatory domain whose phosphorylation initiates channel opening (*5*, *6*). More than 2000 mutations in the CFTR gene have been described to date of which more than 700 are disease-causing (http://cftr2.org). The most prevalent mutation (~80% of patients) is a F508 deletion in NBD1 (*3*), which interferes with CFTR folding at the endoplasmic reticulum (ER) by reducing NBD1 thermal stability and damaging the NBD1-MSD2 interface (*7*, *8*). Misfolded CFTR is prematurely degraded by the ER-associated degradation machinery, thus strongly reducing CFTR trafficking to the plasma membrane (PM). The discovery of pharmaco-chaperones (“CFTR correctors”), small molecules that bind to distinct sites on F508del-CFTR and promote its correct folding and trafficking, has revolutionized CF treatment (*3*, *9*). On the basis of their additive behavior in rescuing folding-deficient CFTR and based on their different binding sites, CFTR correctors can be grouped into three types (*7*, *10*, *11*) with a developing nomenclature. Type I correctors [lumacaftor (LUM), tezacaftor (TEZ), and galicaftor (GAL); all with highly similar structures] bind to a recently defined binding site within MSD1 and stabilize this domain (*12*–*15*) with allosteric effects on overall F508del-CFTR folding. Type I correctors show low efficacy in vitro and in humans as monotherapy (*16*–*19*). Nonclinical type II correctors [corrector 4a (C4a)] are thought to bind to the NBD2 domain to promote F508del-CFTR folding and trafficking (*20*), although their binding site has not been identified yet. The most recently described corrector type, represented by elexacaftor (ELX) and bamocaftor (BAM) that have very similar chemical structures (*16*, *17*), has been categorized as “type III” and was initially thought to bind to and stabilize NBD1 (*7*, *10*, *11*). However, the recently discovered binding site at an interface between the N-terminal lasso domain and MSD2-transmembrane (TM)–10/11 helices rather suggests that these correctors assist in the assembly of MSD1 and MSD2 (*21*). The classification of corrector types (types I, II, and III) that is based on their chronological appearance is followed throughout this manuscript. Type III correctors can have higher efficacies than type I and type II correctors, and when combined with type I correctors, F508del-CFTR folding and trafficking are rescued to ~50% of normal (TEZ + ELX) (*10*, *11*). The most recently approved therapy, Trikafta, combines type III corrector ELX and type I corrector TEZ together with a channel opener ivacaftor (IVA) and causes a substantial improvement in lung function in F508del patients (*16*, *17*, *22*, *23*). The clinical efficacy of Trikafta is superior to the previous combinations of LUM and IVA (Orkambi) or TEZ and IVA (Symdeko), suggesting that cotreatment with multiple correctors of different types improves correct trafficking of folding-deficient CFTR (*22*, *23*), through cooperative pharmaco-chaperoning via the different CFTR binding sites (*11*). Considering the only partial rescue of CFTR function—and the associated persistent abnormalities in mucus properties, airway inflammation, infection, and lung clearance index (*24*, *25*)—even after combining the known corrector types, the identification of highly effective corrector types, with new binding sites and complementary modes of action, is warranted.

Here, we describe macrocyclic CFTR correctors from our drug discovery program, which achieved wild-type (WT)-like folding efficiency of F508del-CFTR through a novel type IV correction mechanism. The corrected F508del-CFTR chloride current in reconstituted CF patient–derived bronchial epithelium matched currents of non-CF controls. Using photo-cross-linkable macrocyclic probes, CFTR peptide mapping, site-directed mutagenesis, and molecular modeling, a cavity between CFTR lasso helix-1 (Lh1) and MSD1-TM1/2 was identified as the most likely macrocycle binding site. This site is distinct from reported binding sites of type I, II, and III correctors, which is consistent with the additive behavior with known correctors. We propose that type IV correctors cotranslationally stabilize Lh1-MSD1-MSD2 interactions, thus drastically increasing the folding efficiency of mutated CFTR. Accordingly, type IV correction also rescued many other CFTR folding mutations including ones resistant to TEZ + ELX treatment, demonstrating promising therapeutic potential for this novel corrector type.

## RESULTS

### Type IV correctors robustly restore F508del-CFTR trafficking and behave additively with type I, type II, and type III correctors

**Fig. 1.**
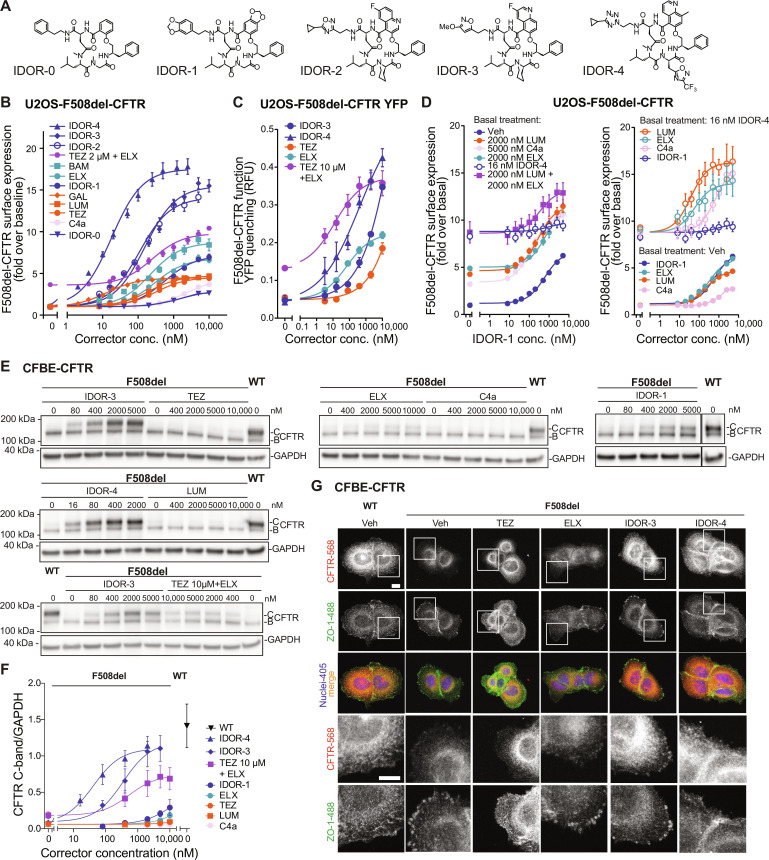
Type IV correctors restore F508del-CFTR trafficking to the cell surface and behave additively with type I, type II, and type III correctors. (**A**) Structures of the macrocyclic type IV correctors used in this study. (**B**) F508del-CFTR cell surface expression in U2OS cells after overnight treatment with different concentrations of the indicated correctors (*n* ≥ 4). (**C**) F508del-CFTR function (YFP quenching assay) after 24-hour treatment of U2OS-F508del-CFTR-YFP cells with different concentrations of the indicated correctors in the acute presence of 0.1 μM forskolin and 2 nM IVA (*n* = 2). (**D**) Left: F508del-CFTR cell surface expression in U2OS cells after overnight treatment with different concentrations of IDOR-1 (*n* = 3) in the presence of vehicle (veh) or a maximally effective concentration of type I corrector (LUM), type II corrector (C4a), type III corrector (ELX), type I + type III correctors (LUM + ELX), or a submaximally effective concentration of IDOR-4. Right: F508del-CFTR cell surface expression in U2OS cells after overnight treatment with different concentrations of type I corrector (LUM), type II corrector (C4a), type III corrector (ELX), or type IV corrector (IDOR-1) (*n* = 3) in the presence of vehicle or a submaximally effective concentration of type IV corrector (IDOR-4). (**E**) Immunoblot analysis of CFBE41o^−^ cells expressing F508del-CFTR or WT-CFTR treated for 24 hours with different concentrations of the indicated correctors and probed for CFTR and glyceraldehyde phosphate dehydrogenase (GAPDH). Representative images of three experiments. (**F**) Quantification of the CFTR C-band intensities in (E) normalized for GAPDH (*n* = 3). (**G**) Localization of CFTR (red) and ZO-1 (green) by immunofluorescence confocal microscopy in CFBE41o^−^ cells treated with either dimethyl sulfoxide (DMSO), 2 μM IDOR-3, 2 μM IDOR-4, or 10 μM and the other indicated correctors for 24 hours. Nuclei appear blue. Scale bar, 10 μm. Data in (B), (C), (D), and (F) are means ± SEM of the indicated number (*n*) of independent experiments. See also figs. S1 and S2.

To identify CFTR correctors with novel chemotypes and a complementary mode of action, we performed a high-throughput screening (HTS) campaign on the Idorsia compound library to discover compounds that increased F508del-CFTR surface expression after overnight treatment and behaved additively with the known corrector types. We used the commercially available U2OS cells expressing F508del-CFTR (DiscoverRx) in which cell surface-residing F508del-CFTR is quantified via the β-galactosidase fragment complementation principle (pharmacotrafficking assay). A total of 67,772 compounds from the Idorsia chemical library were screened at 10 μM with a *z*′ value of 0.79. After applying a cutoff of >13% activity versus the *E*_max_ of LUM (3 μM LUM), 129 primary hits were obtained. After hit confirmation on F508del-CFTR and counterscreening using U2OS cells expressing a different mutated trafficking-defective membrane protein (β_2_-adrenergic receptor ADRB2; DiscoveRx), 83 hits were left (0.12% hit rate). Their potency and efficacy were characterized in the F508del-CFTR pharmacotrafficking assay. Four hits had half effective concentration (EC_50_) values <2 μM and an *E*_max_ > 50% versus LUM *E*_max_ (for statistics, see fig. S1A). Among these was our starting point, 17-membered macrocycle IDOR-0 (Fig. 1A). From this hit during our drug discovery program, the macrocycles IDOR-1 to IDOR-4 (Fig. 1A) were developed and represent a selection of mechanistically novel CFTR correctors. These macrocycles were compared with type I, II, and III correctors in the pharmacotrafficking assay. All correctors increased F508del-CFTR surface expression with different potencies and maximal intrinsic efficacies (Fig. 1B and fig. S1B). The type I correctors (TEZ, LUM, and GAL) and the type II corrector C4a increased F508del-CFTR surface expression maximally four- to fivefold over baseline, whereas the type III correctors (ELX and BAM) increased surface expression maximally seven- to ninefold. In contrast, the selected macrocycles achieved maximal surface expression of 3-, 7-fold (IDOR-0 and IDOR-1) to 14-, 15-, and 18-fold (IDOR-2, IDOR-3, and IDOR-4), respectively. Macrocycle potency ranged between EC_50_ = 1600 nM (IDOR-0) and EC_50_ = 18 nM (IDOR-4), which was up to 10-fold more potent than either TEZ, LUM, BAM, or ELX (Fig. 1B and fig. S1B). ELX, titrated onto a maximally effective concentration of TEZ, reached in combination an *E*_max_ of 10-fold over baseline demonstrating the expected additivity for type I and type III correctors. Corrector activities were fully confirmed by classical anti-CFTR immunoblotting (C-band) in the same samples (fig. S1, C and D). Furthermore, none of the CFTR correctors promoted trafficking of misfolded β_2_-adrenergic receptor ADRB2^W158A^ (fig. S1E), which excludes unspecific effects of correctors on ER quality control (QC).

F508del-CFTR function was assessed in yellow fluorescent protein (YFP) quenching assays (*26*), in the presence of forskolin and the CFTR potentiator IVA (2 nM, ~EC_80_): Overnight treatment of U2OS-F508del-CFTR cells expressing a halide-sensitive YFP with IDOR-3 or IDOR-4 strongly increased F508del-CFTR function (Fig. 1C and fig. S1G). ELX and TEZ were ~2.5- to 6-fold less efficacious, confirming the efficacy ranking of the pharmacotrafficking assay. Again, TEZ + ELX behaved additively. Responses were blocked by the CFTR inhibitor I-172 (fig. S1H), and activity was lower in the absence of IVA (fig. S1F). Some CFTR correctors have acute inhibitory [e.g., GLPG-2737 (*27*)] or copotentiating effects [ELX; (*21*, *26*, *27*)] on CFTR currents. In human embryonic kidney (HEK) 293 cells expressing the gating mutant G551D-CFTR, incubation (10 min) with IDOR-3 or IDOR-4 caused copotentiation of IVA activity, similar to the effect of ELX, while a corrector from the company Galapagos (WO2017060874, example no. 331) displayed an acute inhibitory effect (fig. S1J). These acute effects on a CFTR mutant that does not have a trafficking deficit suggest a direct binding of macrocycles to CFTR without inhibiting its activity, similar to the copotentiator effect of ELX (*21*).

To analyze whether these macrocycles represent a new corrector type with a mechanism complementary to existing correctors, we used moderately active IDOR-1 to be titrated onto maximally effective concentrations of selected reference correctors (Fig. 1D, left). Overnight treatment with IDOR-1 increased F508del-CFTR surface expression when used alone, and IDOR-1 was fully additive over its whole concentration range on top of type I corrector LUM, type II corrector C4a, or type III corrector ELX as demonstrated by the strictly parallel upward shift of the IDOR-1 CRCs in the combinations. Only when applied on top of another macrocycle (IDOR-4), IDOR-1 did not achieve further correction (Fig. 1D, left).

A similar lack of additivity was seen when type I corrector LUM was applied on top of another type I corrector TEZ (fig. S2A) or when the type III corrector ELX was titrated onto the type III corrector BAM (fig. S2B). Also, IDOR-1 was additive to the combination of type I corrector LUM and type III corrector ELX (Fig. 1D, left). The more effective IDOR-3 and IDOR-4 (fig. S2C) showed analogous additive behavior at the lower concentrations before reaching the plateau, which likely represents the maximally possible correction level in this assay. In a complementary approach, reference correctors LUM, C4a, and ELX were titrated onto a highly effective concentration of IDOR-4. Also, in this format, full additivity or even synergy was observed for the type I, II, and III correctors, while IDOR-1 displayed no additivity with IDOR-4 (Fig. 1D, right). In conclusion, macrocycles displayed a clearly additive efficacy on top of the known corrector types and should therefore have a new, complementary mode of action and a distinct CFTR binding site.

Next, we performed anti-CFTR immunoblotting in cystic fibrosis bronchial epithelial cell lines (CFBE41o^−^) expressing either F508del-CFTR or WT-CFTR (*28*). Both cell lines express similar mRNA levels of F508del-CFTR or WT-CFTR, and neither macrocycles nor ELX/TEZ affected these levels, which excludes effects on transcription (fig. S2D). Also, macrocycles did not block the proteasome as deduced from unchanged ubiquitinated protein levels (fig. S2, E and F), excluding this unspecific mechanism. IDOR-3 and IDOR-4 concentration dependently induced strong F508del-CFTR C band accumulation (Fig. 1E), reaching ~75% of the levels observed in WT-CFTR–expressing cells, whereas TEZ, LUM, and ELX showed lower efficacies (Fig. 1F) as already observed in the U2OS system. Again, ELX titrated onto a maximally effective concentration of TEZ behaved additively as expected (Fig. 1F). IDOR-3 and IDOR-4 lead to a ~2-fold increase in WT-CFTR C-band (fig. S2, G and H) as well as function (CFBE-WT-CFTR-YFP cells; fig. S2, I and J), suggesting that even the complex folding of WT-CFTR can profit from highly effective pharmaco-chaperones.

Increased F508del-CFTR C-band expression in CFBE cells reflected trafficking to the cell surface as shown by immunofluorescence microscopy. The level of F508del-CFTR located in PM ruffles, leading edges, or cell-cell contacts as defined by the PM protein ZO-1 was higher in cells incubated with IDOR-3 or IDOR-4 as compared to cells treated with TEZ, ELX, or vehicle (which displayed mainly intracellular CFTR staining reminiscent of the ER) and closely resembled the localization of WT-CFTR (Fig. 1G and fig. S2K).

In summary, we have identified a novel type of CFTR corrector characterized by unprecedented efficacy in promoting folding, trafficking, and function of F508del-CFTR and by additivity with the existing corrector types. We classify these macrocycles with complementary mechanism as type IV correctors.

### Type IV correctors rescue F508del-CFTR trafficking and function in reconstituted cystic fibrosis bronchial epithelium

**Fig. 2.**
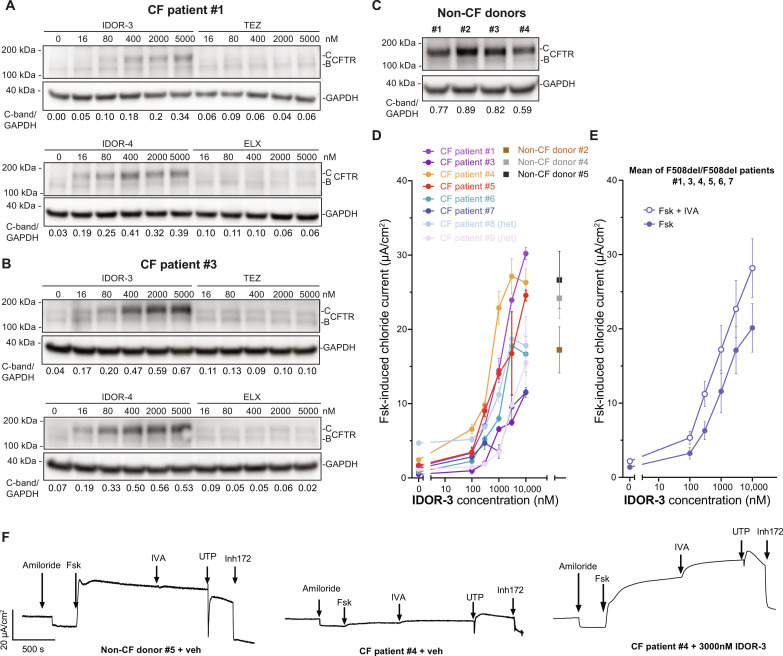
Type IV correctors rescue F508del-CFTR trafficking and function in reconstituted cystic fibrosis bronchial epithelium. (**A** to **C**) CFTR expression (immunoblotting) in reconstituted human bronchial epithelium of two F508del-CFTR homozygous CF patients and four non-CF donors after a 24-hour treatment with the indicated correctors. Representative images of two experiments. The CFTR C-band/GAPDH intensity ratio is represented below every lane. (**D**) Transepithelial forskolin-induced short-circuit currents (Ussing chamber) in reconstituted epithelia of eight CF patients (six F/F, two F/MF) treated overnight with the indicated correctors or vehicle; in addition, tissues from three non-CF donors were treated overnight with vehicle (*n* ≥ 2 measurement per patient/per concentration). (**E**) Mean transepithelial forskolin-induced short-circuit currents for the six F/F patients in (D) with or without acute IVA addition (1 μM). (**F**) Representative examples of raw current traces recorded during a baseline period followed by sequential addition of amiloride, forskolin (Fsk), IVA, uridine triphosphate (UTP), and Inh172 taken from the experiments described in (D) and (E). Data in (D) and (E) are means ± SEM. See also fig. S3. F/F F508del/F508el. F/MF, F508del/minimal function.

Type IV correctors were analyzed in reconstituted human bronchial epithelia derived from biopsies of CF and non-CF donors and cultivated at air-liquid interface (ALI). In tissues from three CF patients (all F508del-F508del), IDOR-3 and IDOR-4 concentration dependently increased CFTR C-band formation (Fig. 2, A and B, and fig. S3A) reaching levels (CF patients #2 and #3) matching the four non-CF donor controls (Fig. 2C). The effects of TEZ and ELX were inferior and difficult to quantify, as reported previously (*17*).

To measure recovery of CFTR function, trans-epithelial chloride currents were measured in Ussing chambers using reconstituted tissue from some of the same and some additional donors (six F508del/F508del, two F508del/minimal function, and three non-CF). IDOR-3 concentration dependently restored forskolin-induced chloride currents to densities observed in the non-CF tissues with some potency and efficacy differences between donors (Fig. 2D), even in the absence of a potentiator. Acute IVA addition resulted in an upward shift of the IDOR-3 concentration-response curve [Fig. 2 (E and F) and fig. S3B for individual patients]. IDOR-4 was tested in reconstituted epithelia of three CF donors with results similar to IDOR-3 but further improved potency (CF patient #1, #4, and #5; fig. S3B). TEZ and ELX, tested on CF patients #3 and #6, showed considerably lower efficacies compared to IDOR-3 (fig. S3B). TEZ (15 μM) + ELX (10 μM) were tested in cultures of homozygous CF patient #6 and behaved additively. Furthermore, TEZ + ELX were also tested in cultures of heterozygous CF patient #8 showing moderate activity. Together, these data using primary reconstituted CF bronchial epithelia confirm the high efficacy of type IV correctors in promoting F508del-CFTR trafficking and function.

### Type IV correctors restore F508del-CFTR folding efficiency in the ER beyond WT levels

**Fig. 3.**
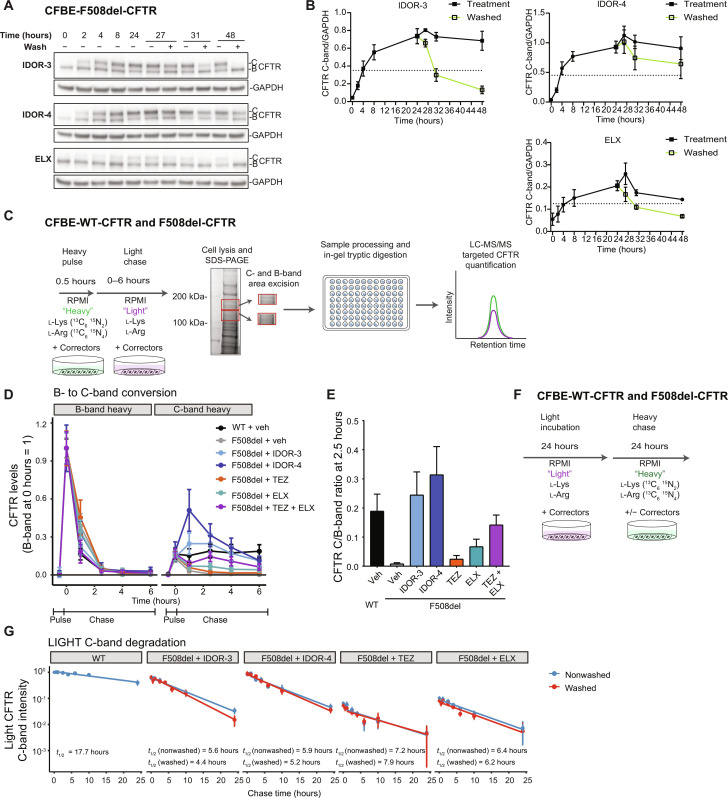
Type IV correctors restore F508del-CFTR folding efficiency in the ER beyond WT levels. (**A**) F508del-CFTR correction kinetics in CFBE41o^−^ cells expressing F508del-CFTR treated with 2 μM IDOR-3 or IDOR-4 or 10 μM ELX for indicated durations including wash-out at 24 hours. Representative images of two experiments. (**B**) F508del-CFTR C-band intensities in (A) normalized for GAPDH (*n* = 2). (**C**) Workflow to determine the ER CFTR folding efficiency using SILAC-based heavy pulse light-chase assays in CFBE41o^−^ cells expressing WT- or F508del-CFTR. (**D**) Average normalized peptide intensities of heavy CFTR from B-bands (left) and C-bands (right) of cells labeled with a 30-min pulse of heavy amino acids and chased with light amino acids. Cells were treated with DMSO (*n* = 5), 2 μM IDOR-3 (*n* = 4), 2 μM IDOR-4 (*n* = 2), 10 μM TEZ (*n* = 3), or 10 μM ELX or TEZ + ELX (10 μM each) (*n* = 3) during both pulse and chase periods. Per treatment, peptide intensities at the different time points were normalized to the corresponding maximal heavy B-band intensity occurring directly after the pulse. (**E**) Folding efficiency determined as average intensity ratio of heavy C-band [2.5-hour chase in (D)] and heavy B-band (directly after pulse) for the different treatments in (D). (**F**) Workflow to determine CFTR stability after folding. (**G**) CFTR C-band half-life with and without corrector wash-out: Cells were incubated 24 hours with light amino acids in the presence of either DMSO (*n* = 4), 2 μM IDOR-3 (*n* = 2), 2 μM IDOR-4 (*n* = 2), 10 μM TEZ (*n* = 2), or 10 μM ELX (*n* = 2) and then chased with heavy amino acids. Correctors were present during the chase (blue lines) or washed-out before the chase (red lines). Half-life was determined by linear regression of the logarithmic data. Data in (B), (D), (E), and (G) are means ± SEM of the indicated number of independent experiments. See also fig. S4. MS/MS, tandem mass spectrometry.

Next, we analyzed the correction kinetics in CFBE cells expressing F508del-CFTR (Fig. 3, A and B). IDOR-3, IDOR-4, and ELX induced C-band increases, reaching steady state after ~8 hours. Correction by IDOR-3 and ELX was reversible upon wash-out with a half-life of ~4 to 6 hours. The effect of IDOR-4 was less reversible possibly due to incomplete wash-out. Thus, the continued presence of correctors is needed to yield stable F508del-CFTR levels.

To analyze whether type IV correctors work via increasing CFTR folding efficiency in the ER (*7*, *11*, *29*), we developed a metabolic pulse-chase assay using stable isotope labeling of amino acids in cell culture (SILAC) (*30*). Proteins in CFBE cells were labeled using a 30-min pulse of medium containing heavy isotopologs of arginine and lysine, followed by a chase in medium containing light amino acids (Fig. 3C). Correctors at maximally efficacious concentrations were present during pulse and chase phases. Cells were then lysed at various time points, and proteins were separated by SDS–polyacrylamide gel electrophoresis (SDS-PAGE). Gel regions containing CFTR B- and C-bands were excised separately, and trypsin was digested and analyzed by high-resolution mass spectrometry (Fig. 3C and fig. S4, A to C). The pulse resulted in heavy labeling of ~30% (WT-CFTR) or ~50% (F508del-CFTR) of the immature CFTR B-pool (fig. S4D). In all conditions, the heavy B-pool disappeared within 2.5 hours of chase (Fig. 3D and fig. S4D), being degraded or matured into the C-pool. Similar to previous studies using recombinant overexpression systems (*7*, *11*, *29*), only a fraction (~19%) of the initial heavy B-pool of WT-CFTR was converted to a heavy C-pool (2.5-hour chase), while <1% of the heavy B-pool of F508del-CFTR was converted at this time point (Fig. 3, D and E). TEZ or ELX correction of F508del-CFTR increased B-to-C conversion rates to 2.4 or 6.7%, respectively, while IDOR-3 or IDOR-4 resulted in conversion rates of 24 to 31%, which were thus higher than observed for WT-CFTR. The TEZ + ELX combination yielded a conversion rate of 14%, i.e., the two correctors behaved additively. We conclude that type IV correctors elevate F508del-CFTR ER folding efficiency to or even beyond WT levels, i.e., fully compensate the F508del-induced folding defect.

To determine how correctors affected stability of already folded F508del-CFTR, we labeled the whole CFTR pool with light amino acids and performed a chase with heavy amino acids. Correctors at their maximally effective concentrations were either present during both pulse and chase periods or washed out after the pulse (Fig. 3F). For WT-CFTR, the light C-pool decayed slowly [half-time (*t*_1/2_) = 18 hours], whereas the light C-pools of F508del-CFTR corrected with TEZ, ELX, IDOR-3, or IDOR-4 decayed faster (*t*_1/2_ of ~5 to 6 hours), irrespective of corrector type and presence, suggesting that none of the correctors were efficient CFTR stabilizers (Fig. 3G and fig. S4E, top). Wash-out of the correctors (except IDOR-4, still showing 50% of heavy C-pool) was confirmed by the lack of new heavy C-pool formation in the washed conditions (fig. S4E, bottom). Similar results were obtained in reconstituted bronchial epithelium (fig. S4, F and G) where the WT-CFTR C-pool displayed a *t*_1/2_ of ~15 hours, while the IDOR-3– and IDOR-4–corrected F508del-CFTR C-pool had a *t*_1/2_ of ~5 to 7 hours. For TEZ- and ELX-treated samples, *t*_1/2_ values could not be determined as the signal was too low. In contrast to previous techniques, which lack wash-out controls and use cycloheximide to block protein neosynthesis (*7*, *11*), this highly sensitive pulse-chase approach showed that neither type III, I, nor the new type IV correctors alter F508del-CFTR postfolding stability. However, since type IV correctors promote F508del-CFTR ER folding to up to 170% of WT efficiency, a steady-state F508del-CFTR expression of almost WT levels is reached (Fig. 1F).

### Type IV correctors overcome thermal instability during F508del-CFTR synthesis and require MSD1-NBD1-R-MSD2 as minimal substrate

**Fig. 4.**
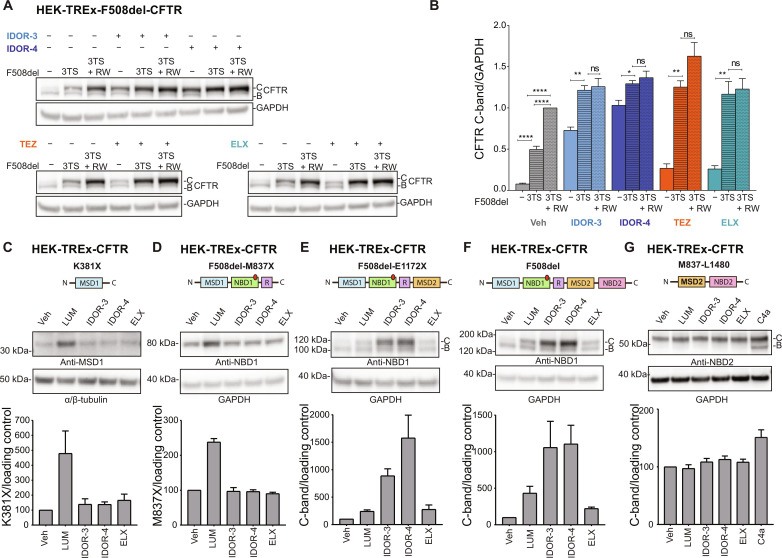
Type IV correctors restore native folding by overcoming thermal instability during F508del-CFTR synthesis and require MSD1-NBD1-R-MSD2 as minimal substrate. (**A**) CFTR correction (immunoblotting) in HEK-TREx cells expressing either F508del-CFTR or thermostabilized F508del-CFTR-G550E.R553M.R555K (3TS) or thermostabilized and interface-stabilized F508del-CFTR-G550E.R553M.R555K.R1070W (3TS + RW). Cells were treated for 48 hours with DMSO or maximally effective concentrations of either IDOR-3 (2 μM), IDOR-4 (0.4 μM), TEZ (2 μM), or ELX (0.4 μM). Representative images of three experiments. (**B**) CFTR C-band intensities in (A) normalized for GAPDH and further normalized to the C-band intensity of vehicle-treated F508del-CFTR-3TS + RW cells (*n* = 3). (**C** to **G**) Levels of truncated CFTR constructs (immunoblotting) expressed in HEK-TREx cells after 40 hours of treatment with vehicle or maximally effective concentrations of either LUM (2 μM), IDOR-3 (2 μM), IDOR-4 (0.4 μM), ELX (0.4 μM), or C4a (10 μM). Detection antibodies target either MSD1 (C), NBD1 (D to F), or NBD2 (G). Intensities of CFTR fragment bands are shown in the graphs [C-band for (E) to (G)], normalized for the loading controls [*n* = 3 for (C) to (F), *n* = 4 for (G)]. Data in (B) to (G) are means ± SEM of the indicated number of independent experiments. In (B), a one-way analysis of variance (ANOVA) test was performed between the three different mutants within every compound treatment group; ns: nonsignificant: *P < 0.05; **P < 0.01; ****P < 0.0001. See also fig. S5.

We further dissected type IV correction mechanistically. Rescue of ER folding efficiency to WT levels can be achieved by simultaneous correction of the thermal and interface instability in F508del-CFTR, e.g., by introducing suppressor mutations that thermally stabilize NBD1 (i.e., G550E.R553M.R555K or “3TS”) in combination with mutations that increase NBD1-MSD2 interface interactions (R1070W or “RW”) (*8*, *31*–*33*). HEK-F508del-CFTR.3TS cells expressed sixfold more C-band CFTR than HEK-F508del-CFTR cells, and the addition of the RW mutation yielded a further 2-fold increase for an overall 12-fold increase (Fig. 4, A and B). We applied the correctors at maximally effective concentrations as assessed for this specific cell type and serum concentration (fig. S5, A and B). In F508del-CFTR cells, IDOR-3/IDOR-4 induced 9-/12-fold increases in C-band expression (Fig. 4, A and B), i.e., IDOR-4 had the same efficiency as the combined introduction of 3TS and RW mutations. Combining IDOR-3 or IDOR-4 with 3TS mutations only marginally improved C-band expression (~1.5-fold), and the introduction of the RW mutation had no additional effect. In contrast, TEZ and ELX strongly increased C-band expression (~4-fold) after introduction of 3TS mutations, indicating that TEZ and ELX do not rescue thermal instability (Fig. 4, A and B) (*8*, *34*). Together, type IV correctors have the ability to fully overcome the F508del-CFTR folding defects yielding efficiencies comparable to the suppressor mutations that remedy both thermal and interface instability during folding.

To pinpoint the CFTR protein domains involved in type IV correction, we generated HEK cells expressing CFTR fragments of increasing size (Fig. 4, C to G) as previously described (*35*). Expression of MSD1 (K381X-CFTR, Fig. 4C) increased fivefold after LUM treatment in agreement with the recently published binding site and mode of action of type I correctors, which bind to and increase the stability of MSD1 (*12*–*15*). IDOR-3, IDOR-4, and ELX had no effect. Similar results were obtained for the MSD1-NBD1-R fragment (F508del-M837X-CFTR, Fig. 4D). A clear change was observed for the MSD1-NBD1-R-MSD2 fragment (F508del-E1172X-CFTR, Fig. 4E), which was corrected and glycosylated to produce a C-band by treatment with LUM or ELX (2- to 3-fold over baseline) and especially IDOR-3 or IDOR-4 (10- to 15-fold over baseline). Similar effects were observed for full-length F508del-CFTR (Fig. 4F). None of these correctors affected expression of the MSD2-NBD2 fragment (M837-L1480-CFTR, Fig. 4G), while type II corrector C4a, proposed to promote NBD2 folding (*36*), increased the expression of its glycosylated and nonglycosylated forms. These data confirm that type I correctors act on MSD1, and type II correctors act on NBD2. They demonstrate that type IV and type III correctors require an almost full-length CFTR substrate including MSD2, likely because they promote the challenging assembly of MSD2 with the preceding domains.

### Type IV correctors bind to the CFTR lasso domain

**Fig. 5.**
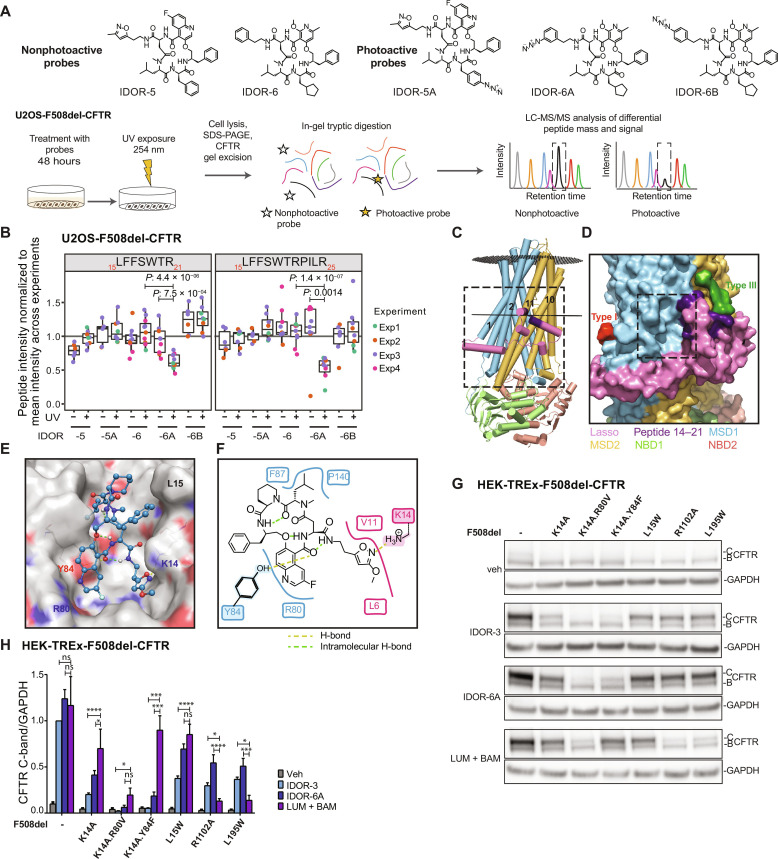
Type IV correctors bind to the CFTR lasso domain. (**A**) Type IV corrector binding site determination: photoactive probes, workflow. (**B**) Abundance of CFTR peptides _15_LFFSWTR_21_ and _15_LFFSWTRPILR_25_ from cells treated with non-photoactive IDOR-5 or IDOR-6 or photoactive IDOR-5A, IDOR-6A, or IDOR-6B and then exposed or not to UV light (5 min). Each peptide signal was normalized to the CFTR amount in that sample (four independent experiments, same color dots represent technical replica). Significant *P* value after one-sided *t* test (not adjusted for multiple testing) only for IDOR-6A versus IDOR-6 (with UV) and IDOR-6A with UV versus without UV. (**C**) F508del-CFTR in the phosphorylated open state (PDB: 8EIQ). Cross-linked lasso domain peptide (including K14) _14_KLFFSWTR_21_ in purple. TM1, TM2, TM10, and TM11 are highlighted. (**D**) Cavity adjacent to cross-linked peptide facing TM1/2 and able to accommodate active macrocycles. Type I/III correctors (21) in red/green. (**E** and **F**) Three- and two-dimensional views of bound IDOR-3 as determined by docking studies. Polar amino acid side chains (E) in blue, red, and yellow. H-bonds between IDOR-3 and CFTR as yellow dashed lines. IDOR-3 intramolecular H-bonds as green dashed lines. In (E), Y84 and K14 side chains are highlighted. (**G**) Corrector activities after site-directed mutagenesis of key amino acids in the proposed type IV corrector binding site: Cells expressing F508del-CFTR containing one/two additional mutations in the site (K14A; K14A.R80V; K14A.Y84F; L15W), the ELX site (R1102A), or the LUM site (L195W) were treated (40 hours) with indicated correctors (2 μM). Representative images from at least *n* = 4 to 10 independent experiments per mutant and corrector. (**H**) Quantification of C-band intensities in (G), GAPDH-normalized. Data are means ± SEM. One-way ANOVA test per mutant for the different compound treatments, comparison between [LUM + BAM] and the two macrocycles is shown. *P < 0.05; ***P < 0.001; ****P < 0.0001. See also figs. S6 and S7.

To covalently label the type IV corrector binding site, three photo-activatable aryl-azide macrocycle probes (IDOR-5A, IDOR-6A, and IDOR-6B) were developed from non-azide parent molecules IDOR-5 and IDOR-6 (Fig. 5A). These five compounds were designed with ease-of-synthesis considerations and showed pharmaco-chaperone activity comparable to IDOR-3 and IDOR-4 (fig. S6, A to C). In buffer containing primary amines (tris), the azide-containing probes were completely turned over within 5 min of ultraviolet (UV) illumination demonstrating their UV-inducible reactivity (fig. S6D). U2OS-F508del-CFTR cells treated with the three photo-activatable probes or the two non-azide controls were exposed to UV illumination or not. Proteins were separated by SDS-PAGE, and the CFTR-containing gel regions were excised and trypsin-digested for liquid chromatography–mass spectrometry (LC-MS) analysis (Fig. 5A). In four independent experiments, an average of 73 CFTR peptides were detected corresponding to 53% of the protein (fig. S6, E and F). Only 2 of these 73 peptides were significantly (with multiple testing correction) reduced under UV exposure when comparing each photoactive probe with its corresponding non-active parent macrocycle: Compared to parent IDOR-6, IDOR-6A reduced by ~50% the abundance of the overlapping peptides _15_LFFSWTR_21_ (*P*_adj_ = 0.0002) and _15_LFFSWTRPILR_25_ (*P*_adj_ = 0.00001) (Fig. 5B and fig. S6G). No other peptide showed significant down-regulation (fig. S6, G and H). The results for these two peptides without multiple testing correction confirmed significant reductions for IDOR-6A versus IDOR-6 (with UV) and, in addition, for IDOR-6A with UV versus without UV and showed no differences for the other two aryl-azides IDOR-6B and IDOR-7A (Fig. 5B). Thus, cross-linking of IDOR-6A occurred between amino acids 14 and 21 (K14 is recognized by trypsin), i.e., at the N terminus of CFTR, within Lh1 (Fig. 5C). Neither type I, III, nor IV correctors could rescue F508del-CFTR in which amino acids 14 to 21 had been deleted (fig. S7A), suggesting that amino acids 14 to 21 are crucial for the CFTR folding process. In contrast, deletion of amino acids 14 to 21 in the isolated MSD1 domain led to an increased MSD1 expression (fig. S7B), suggesting a destabilizing effect of amino acids 14 to 21 on the isolated domain itself. These data indicate that type IV correctors interact with a sequence in Lh1 that plays an essential role in folding full-length CFTR.

Considering the macrocycle size, two binding sites adjacent to peptides 14 to 21 were conceivable (Fig. 5, C and D). One site faces MSD2-TM11 (Fig. 5D) and is identical to the recently described binding site for type III correctors, here shown with ELX bound (*21*). The remaining other site faces MSD1-TM1/2 (Fig. 5D, box) and is a cavity at the membrane-cytosol interface that can accommodate active macrocycles such as IDOR-3 with high shape complementarity and involving polar interactions with at least two polar amino acids (K14 in Lh1 and Y84 in MSD1) as determined by molecular docking (Fig. 5E). This is exemplified for IDOR-3, in which the isoxazole moiety forms an H-bond with the side chain amine of K14, and one central-ring carbonyl forms an H-bond with the hydroxy group of the Y84 side chain. The other interactions are lipophilic and based on optimized shape complementarity. Thus, IDOR-3 is proposed to act as a bridge between MSD1-TM1/2 and Lh1. All three nonmethylated macrocyclic amides are involved in intramolecular H-bonds, placing the macrocycle in a favorable, low energy conformation (Fig. 5, E and F). This binding mode hypothesis was transferable to the other active macrocycles and used to rationalize why IDOR-6A was the only aryl-azide that could be cross-linked to CFTR: In IDOR-5A, the aryl-nitrene (activated intermediate) is too distant from the CFTR surface (fig. S7C). In IDOR-6A, the aryl-*meta*-nitrene is optimally placed to covalently bind to the K14 amine (fig. S7, E and F), while the aryl-*para*-nitrene in IDOR-6B is not (fig. S7D).

Next, this site was explored by introducing single or double mutations to the three polar amino acids K14, Y84, and R80 within F508del-CFTR to either disrupt polar interactions (K14A, K14A.R80V, and K14A.Y84F) or occlude part of the site (L15W). Known mutations abolishing type III (R1102A) and type I (L195W) correction were introduced as controls (*12*, *21*). Cells were then treated (40 h) with different corrector types and combinations at their maximally effective concentrations as assessed for this specific cell type and serum concentration (fig. S5, A and B). Without correctors, CFTR mutants were expressed only as B-bands (Fig. 5, G and H). The double and triple mutants generally displayed slightly lower correctability as compared to F508del-CFTR (fig. S7, G and H). Our analysis of the different mutations deliberately focused on comparing the activity of macrocycles (IDOR-3 and IDOR-6A) versus (LUM + BAM = type I + type III correction), because [LUM + BAM] and macrocycles reached similar correction of F508del-CFTR in this cell type (Fig. 5, F and G) allowing for clear interpretation of mutation effects: F508del.L195W-CFTR and F508del.R1102A-CFTR were only weakly corrected by [LUM + BAM] compared to the macrocycles (~3- to 5-fold lower), as expected for mutations disrupting type I or III corrector binding. F508del.L15W-CFTR showed little difference in correction by macrocycles versus [LUM + BAM]. In contrast, F508del.K14A-CFTR displayed a weaker correction by macrocycles (two- to fourfold), as compared to [LUM + BAM]. A notable selective 5- to 10-fold loss of correction for macrocycles versus [LUM + BAM] was observed in the F508del.K14A.Y84F triple mutant. The F508del.K14A.R80V triple mutant was barely correctable by any corrector, likely due to loss of an essential intraprotein salt bridge involving R80. These data (Fig. 5, G and H) and further data in the Supplementary Materials (fig. S7, G and H) strongly suggest that type IV correctors bind in the cavity between Lh1 and MSD1-TM1/2, interacting with the polar amino acids K14 and Y84.

### Type IV correctors address non-F508del CFTR folding mutations

**Fig. 6.**
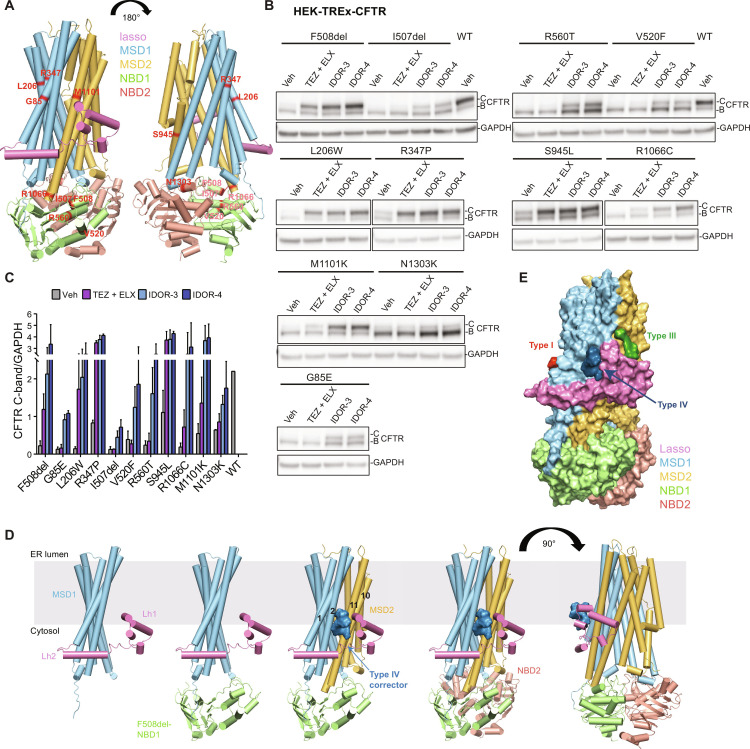
Type IV correctors address non-F508del CFTR folding mutations. (**A**) Cylinder view of the WT-CFTR channel in the phosphorylated-open state (PDB: 6MSM) with the amino acids of the corresponding mutations colored in red. (**B**) CFTR correction (immunoblotting) in HEK-TREx cells expressing 11 different class II folding mutations [see (A) and (B)] and treated for 48 hours with DMSO or maximally effective concentrations of IDOR-3 (2 μM) or IDOR-4 (0.4 μM) or a combination of TEZ (2 μM) + ELX (0.4 μM). Representative images from two experiments. (**C**) CFTR C-band intensities in (B) normalized for GAPDH (*n* = 2). Data represent mean ± SEM of two independent experiments. (**D**) Model depicting type IV corrector binding to the Lh1-MSD1-MSD2 interface to support the challenging interdigitation of transmembrane helices which occurs late in translation. (**E**) Model of simultaneous type I, III, and IV corrector binding to CFTR. See also fig. S5.

The observed site of action of type IV correctors which is distant from the actual F508 deletion suggested that other *CFTR* folding mutations might be rescued. We expressed the 10 most frequent CF-causing *CFTR* folding mutations (https://cftr2.org) (Fig. 6A and fig. S7I). Using maximally effective concentrations (fig. S5, C and D), we tested IDOR-3, IDOR-4, and [TEZ + ELX] (stand-alone TEZ or ELX treatment showed no/little correction in many mutants, fig. S5, C and D). [TEZ + ELX] were highly effective in L206W, R347P, and S945L, moderately effective in M1101K and R1066C, and not effective in G85E, I507del, R560T, V520F, and N1303K (Fig. 6, B and C, and fig. S5, C and D). In contrast, macrocycles were highly effective in all mutants except N1303K where only minor activity was detectable (Fig. 6, B and C), showing that the allosteric mechanisms promoting MSD1/2-NBD1 folding only weakly extend to the C-terminal NBD2. Together, type IV correction can address many non-F508del *CFTR* folding mutations including the currently not treatable I507del, R560T, V520F, and R1066C, demonstrating an unprecedented potential for this novel corrector mechanism.

## DISCUSSION

CFTR modulators have significantly improved life quality and expectancy for many CF patients. Since single-corrector treatments showed limited effects, the current standard of care for F508del-CFTR patients combines type I corrector TEZ and type III corrector ELX to mediate—together with the potentiator IVA—a substantial improvement of CFTR function. Current consensus is that CFTR correctors must act in concert to achieve clinically effective correction; however, CFTR folding and trafficking are still not recovered by more than ~50%. This deficit warrants the search for correctors with novel, complementary mechanisms, to be used in addition to existing corrector types or even as highly effective stand-alone treatments. Compared to type I, II, and III correctors, the macrocyclic type IV correctors from our drug discovery program display unprecedented efficacy in promoting folding and trafficking of F508del-CFTR in cell lines and reconstituted patient tissue. In addition to having therapeutical potential to address the F508del folding mutation as well as many others, type IV correction also sheds more light onto the CFTR folding process because the interdomain macrocycle binding site pinpoints a CFTR region highly relevant to its folding.

Three complementary approaches using photo-activatable probes, molecular modeling/docking, and site-directed mutagenesis resulted in the identification of a highly probable binding site of type IV correctors. Cross-linking yielded only a single CFTR peptide pair that was consistently reduced under UV exposure by only one (the aryl-*meta*-azide) of the three cross-linkable probes and suggests a highly specific macrocycle interaction with CFTR peptides14 to 21. The N terminus of CFTR (amino acids 1 to 80) is highly conserved among species and ABC-C transporters (*37*) and is an important contributor to folding. Lh2 (amino acids 46 to 61) interacts with MSD1-TM1/2/3/4 and the N terminus of NBD1 to create a first important domain assembly unit (*13*). The function of Lh1 (amino acids 11 to 29) remained largely unknown until the recent disclosure of the cryo–electron microscopy structure of F508del-CFTR bound to ELX, TEZ, and IVA in which ELX is positioned in a void between Lh1 and MSD2-TM11 with Lh1 packing against MSD1-TM1/2 and MSD2-TM10/11 (*21*, *38*). Our study highlights a central role of Lh1 in promoting folding of mutated and probably also WT-CFTR: The deletion of amino acids 14 to 21 abolishes the folding capacity of F508del-CFTR in the presence of any corrector (fig. S7A), while the same deletion in the isolated MSD1 strongly increases domain expression (fig. S7B). This contrasting behavior suggests a central chaperoning role of Lh1 in MSD1-MSD2 interdomain assembly likely assisting in the challenging interdigitation of freshly synthesized TM10/11 of MSD2 into the TM1/2/3/6 helix bundle of MSD1. Accordingly, type IV correctors that interact with Lh1 exert their stabilizing effects only if the CFTR sequence spans the region from the N terminus to MSD2 (Fig. 4, C to G), in contrast to type I correctors that promote MSD1 intradomain stability (Fig. 4C) (*12*–*15*, *39*). Thus, it is likely that macrocycles, by binding in the cavity between Lh1 and MSD1-TM1/2, stabilize a late translational intermediate complex of Lh1, MSD1, and MSD2 (Fig. 6D). Type III correctors, according to (*21*) and our data, have a related but not identical mode of action (hence the additivity) by stabilizing Lh1 from the other side which faces MSD2-TM11, but with lower efficacy. We conclude that Lh1-mediated interdomain assembly of MSD1 and MSD2 is crucial for CFTR folding, and pharmaco-chaperones acting on this step—the type III and the more efficient type IV correctors—can allosterically overcome the need of a direct rescue of NBD1 instability during folding (Fig. 4, A and B). The non-overlapping nature of corrector type I, III, and IV binding sites (Fig. 6E) plausibly explains their additive behavior in correcting CFTR folding. Together, characterizing type IV correction has uncovered a short N-terminal amino acid stretch—Lh1—with a central role in CFTR interdomain assembly. Using this highly effective allosteric mechanism, type IV correctors can address many severe folding mutations in MSD1, NBD1, and MSD2 that are not correctable with other corrector types (Fig. 6, A to C) and even slightly promote folding of WT-CFTR.

Steady-state levels of cell surface CFTR are governed by CFTR folding/trafficking efficiency (endoplasmic reticulum) and CFTR postfolding half-life (plasma membrane). Combining the SILAC pulse-chase technique with SDS-PAGE, B/C-band excision and CFTR peptide LC-MS analysis allowed us to precisely quantify these processes and their modulation in CFBE cells. WT-CFTR folding efficiency has been described to range from 20% in recombinant systems (*7*, *11*, *27*) to ~80% in endogenously expressing systems (*40*). The WT-CFTR folding efficiency found in this study (~20%) and the protein half-life (*t*_1/2_ = 18 hours) were in agreement with previous reports using recombinant overexpression systems as were those of F508del-CFTR (<1% folding efficiency) (Fig. 3, D and E). Folding efficiency of the analyzed correctors was between 2 and 31% (Fig. 3, D and E) displaying the same rank order as determined in Western blotting of F508del-CFTR steady-state levels (Fig. 1, E and F). This indicates that the difference in corrector efficacy on steady-state CFTR levels is a consequence of their different CFTR ER folding efficacies reaching more than WT levels with the best type IV correctors (31%). However, no corrector (including type IV correctors) prolonged the stability of already folded F508del-CFTR (*t*_1/2_ ~ 5 hours; Fig. 3G) as determined with our novel pulse-chase technique avoiding cycloheximide and including corrector washout controls. Thus, correctors promote the stability of CFTR folding intermediates during translation but do not address the stability deficit of mature F508del-CFTR. Still, steady-state expression of macrocycle-corrected F508del-CFTR almost reaches WT levels, likely because ER folding efficiencies are increased considerably beyond WT levels. In agreement with this, macrocycles can also improve WT-CFTR steady-state levels and function (fig. S2, G to J), suggesting further investigation for treatment of other CFTR deficit–related respiratory diseases, such as chronic obstructive pulmonary disease.

In conclusion, type IV correction optimally supports an essential intramolecular folding mechanism involving the previously underestimated Lh1 and thus fully normalizes F508del-CFTR folding and trafficking to essentially WT levels, reaching efficacies that are superior to the previously known type I, II, and III corrector mechanisms. Since this unprecedented efficacy is recapitulated in reconstituted CF patient tissue, type IV correctors whose drug-like properties will be described in due course could represent a promising new treatment modality for CF patients with *CFTR* folding mutations. A first type IV corrector has been progressed into preclinical development.

## MATERIALS AND METHODS

### Compounds

LUM was purchased from Astatech, TEZ from Selleckchem, IVA from Combi-Blocks, and C4a from Manchester Organics. All other compounds used in this study were synthetized at Idorsia Pharmaceuticals Ltd. (Allschwil, Switzerland). The synthesis of correctors IDOR-1 to IDOR-4 is described in patent WO2022194399A1.

### Cell line origin and culture

PathHunter U2OS-F508del-CFTR MEM-EA and PathHunter U2OS-ADRB2(W158A) ENDO-EA cells (abbreviated U2OS F508del-CFTR and U2OS ADRB2[W158A]) were obtained from DiscoverX (catalog nos. 93-0987C3 and 93-0986C3, respectively) and were propagated in their culture medium (AssayComplete Cell Culture Kit 103, DiscoverX) according to the manufacturer’s instructions.

Cystic fibrosis bronchial epithelial (CFBE41o^−^) cells stably expressing the human WT CFTR or F508del-CFTR mutant [generated in (*26*)] were a gift from J. P. Clancy (University of Alabama, Birmingham) and cultured on fibronectin-coated plates (FNC coating mix; Enzo) in minimum essential medium with Earl’s salt (Thermo Fisher Scientific) containing 10% of heat-inactivated fetal bovine serum (FBS; Gibco), penicillin (100 U/ml), streptomycin (100 mg/ml) (Thermo Fisher Scientific), and puromycin (2 μg/ml; Sigma-Aldrich).

The human CFTR expressing HEK cell pools with the mutations described below were generated by integrase-mediated homologous recombination of the CFTR sequence into the T-REx HEK293 background (Thermo Fisher Scientific). This expression system allows the tetracycline-inducible expression of genes of interest from one defined insertion site. Parental T-REx-HEK293 cells were cultivated in growth medium: Dulbecco’s modified Eagle’s medium + GlutaMAX-I (#31966, Life Technologies), 10% dialyzed FBS (Gibco, #26400), penicillin (100 U/ml), streptomycin (100 μg/ml), hygromycin B (0.1 mg/ml; Life Technologies), blasticidin (5 μg/ml; Invitrogen, #R210-01), and 0.1 mM nonessential amino acid solution (Invitrogen, #11140-035). Recombinant T-REx-HEK-CFTR cells were generated by lipofectamine transfection (Life Technologies) of plasmids containing mutated versions of CFTR (RefSeq NM_000492) and then cultivated in selection medium [growth medium containing Geneticin (1 mg/ml) (Invitrogen, #10131-027) instead of hygromycin]. The plasmid constructs were synthetized by GENEART (Thermo Fisher Scientific) and designed with the WT *CFTR* intron 5 to 6 (880 nucleotides) to prevent leaky expression issues during the cloning process in bacteria. For evaluation of corrector efficacies on second-site suppressor mutants, the following mutations were introduced into the *CFTR.F508del* gene sequence: G550E.R553M.R555K and G550E.R553M.R555K.R1070W. The following plasmids expressing different truncated versions of *CFTR.F508del* gene were generated: K381X, delF508-M837X, delF508-E1172X, and M837-L1480. For the evaluation of different binding sites by site-directed mutagenesis, the following mutations were introduced individually into the *CFTR.F508del* gene: del14-21, K14A, K14A.R80V, K14A.Y84F, L15W, R1102A, and L195W. Deletion of the peptide stretching amino acids 14 to 21 was also introduced into truncated K381X: del14-21.K381X. To assess corrector capacity to stabilize different CF-causing, class II folding mutants other than F508del, the following mutations were introduced to the *CFTR* WT gene sequence: G85E, L206W, I507del, V520F, R560T, S945L, R1066C, M1101K, N1303K, and R347P. For copotentiation experiments in the YFP-quenching assay, the CF-causing, class III gating mutation G551D was introduced to the *CFTR* WT gene sequence. For the halide-sensitive YFP quenching assay, U2OS F508del-CFTR or TREx-HEK-G551D-CFTR cells were transduced with lentiviral particles rLV.EF1. F46L/H148Q/I152L-YFP (Vectalys, Flash Therapeutics) carrying a mutated halide-sensitive version of *YFP* (*26*). Briefly a suspension of 5,000,000 cells was added to a mix of lentiviral vectors (final concentration 2.7 × 10^9^ transduction units/ml) mixed with 4 μg of polybrene (Sigma-Aldrich). Then, cells were seeded and expanded in their respective growth medium.

### Pharmacochaperone trafficking assay

This assay was adapted from the DiscoverX PathHunter pharmacochaperone trafficking assay, which is based on the enzyme fragment complementation technique. Briefly, the U2OS-F508del-CFTR cell line (DiscoveRx, #93-0987C3) is engineered to coexpress (i) human F508del-CFTR tagged with a Prolink (PK = short β-galactosidase fragment) and (ii) the remainder of the β-galactosidase enzyme [enzyme acceptor (EA)] localized to the PM. Incubation with compounds that increase trafficking of PK-tagged F508del-CFTR to the PM will lead to complementation of the EA fragment to form a functional β-galactosidase enzyme, which is quantified by a chemiluminescence reaction reporting cell surface CFTR expression. The same principle applies to the counterscreen using U2OS-ADRB2(W158A) cells (DiscoveRx, #93-0986C3). Freshly thawed U2OS-F508del-CFTR and U2OS-ADRB2(W158A) were seeded at 3500 or 5000 cells per well, respectively, in 20 μl per well of cell assay medium [McCoy (Life Technologies), 10% FBS, penicillin (100 U/ml), and streptomycin (100 mg/ml)] in a 384 white low-volume plate (Corning). Predilutions of compounds in dimethyl sulfoxide (DMSO) were prepared in a 384-well polypropylene plate using an automated liquid-handling system. Dilution series were then transferred into cell assay medium in a polypropylene 384-well plate (Greiner) to obtain 5× concentrated aqueous working stocks. Five hours after cell seeding, 5× working stocks (5 μl per well) were added to the cells (20 μl per well) for an incubation overnight at 37°C. The next day, after 2 hours at room temperature, the cell plates were incubated with 10 μl of Flash detection reagent (#93-0247, DiscoverX) for 30 min, and chemiluminescence signals were detected with a microplate reader (Synergy MX, Agilent BioTek). Chemiluminescence data were normalized to the signal of basal vehicle-treated cells, and mean chemiluminescence values were converted to concentration-response curves for determination of EC_50_ and *E*_max_ values (GraphPad Prism).

Both above described cell lines [U2OS-F508del-CFTR and U2OS-ADRB2(W158A)] and assays were also used in the high-throughput screening campaign in which compounds were tested at 10 μM concentration with LUM (3 μM) or propranolol (100 nM) serving as 100% controls.

### Reconstituted primary human bronchial epithelial tissues

Reconstituted tissues from CF and non-CF donors were either directly purchased as fully differentiated ALI culture from Epithelix SA (Geneva) for direct experimental use or generated in our laboratories from passage 1 human airway epithelial cells (hAECs; obtained from Epithelix SA) using the differentiation protocols and reagents provided by STEMCELL Technologies (PneumaCult reagent series). In-house generated cultures were derived from three homozygous F508del-CFTR patients: CF patient #1 (EP57AB-CFAB060901, Epithelix, female, 21 years old), CF patient #2 (EP57AB02-CFAB0452, Epithelix, male, 32 years old), and CF patient #3 (#28388-0000450918, Lonza, male, 25 years old); and four non-CF donors with no pathology reported: non-CF #1 (EP51AB-02AB079301, Epithelix, male, 62 years old), non-CF #2 (EP51AB-02AB0834.01 Epithelix, male, 71 years old), non-CF #3, (EP51AB-02AB0839.01, Epithelix, male, 59 years old), and non-CF #4 (CC-2540S-20TL356517, Lonza, female, 48 years old). hAECs were cultured for several days until reaching 90 to 95% confluency in complete PneumaCultExPlus medium. Cells were then washed with phosphate-buffered saline (PBS) and dissociated with ACF enzymatic dissociation solution, followed by addition of ACF enzyme inhibition solution. Cells were seeded onto single Transwell inserts (polyester membranes, Corning, #CLS3470 in 24-well plates, #Corning 3524) sitting in complete PneumaCultExPlus medium (600 μl per well) at a density between 30,000 and 50,000 cells per insert and cultivated submerged for several days. Once cells reached 90 to 95% confluency, medium was removed from the apical side to expose cells to air, while the medium on the basal side was replaced with PneumaCult ALI (600 μl per wellI) maintenance medium. Basal medium was exchanged every 3 days for 4 weeks until full differentiation was reached as indicated by ciliary beating, mucus production, and tight junction formation (ZO-1 staining).

### Treatments with correctors for immunoblotting

For testing concentration-response effects of correctors on rescuing F508del-CFTR C-band expression in U2OS-F508del-CFTR cells, cells were treated as described for the pharmacochaperone trafficking assay. CFBE-F508del-CFTR or CFBE-WT-CFTR were seeded at 140,000 cells per well in a FNC-coated 24-well plate for a day and then incubated for 24 hours with ascending concentrations of correctors diluted in growth medium deprived of FBS but supplemented with 1% human serum albumin (HSA; Sigma-Aldrich, #A1653). The same protocol was used for treatment with proteasome inhibitor MG-132 (Sigma-Aldrich). For CFTR expression kinetics and reversibility upon wash-out, CFBE-F508del-CFTR cells were seeded and treated as above with fixed concentrations of correctors for 2, 4, 8, 24, 27, 31, and 48 hours. At the 24-hour time point, cells for wash-out analysis were washed twice for 10 min with CFBE growth medium and then incubated for 3, 7, or 24 hours in 1% HSA medium without correctors. For all experiments with primary bronchial ALI tissues, correctors were added to the basal medium for 24 hours. For all experiments with isogenic T-REx-HEK cells expressing various CFTR mutants, cells were seeded at 200,000 cells per well in a 24-well plate for a day and then incubated for 40 hours with ascending or fixed concentrations of correctors diluted in growth medium supplemented with tetracycline (10 ng/ml; tetracycline hydrochloride, Sigma-Aldrich, #T7660). For T-REx-HEK cells expressing truncated versions of CFTR, tetracycline (100 ng/ml) was used to ensure sufficient expression levels.

### Immunoblotting

All cells were washed with ice-cold PBS and then lysed for 1 hour on ice with radioimmunoprecipitation assay (RIPA) buffer (Sigma-Aldrich, #R0278) containing 100 mM Na-fluoride, 4 mM Na-orthovanadate, 1 mM phenylmethylsulfonyl fluoride, 1 mM dithiothreitol, and benzonase (100 U/ml; Sigma-Aldrich). Primary bronchial ALI tissues were lysed for 2 hours on ice and with double concentration of benzonase. Samples were mixed with LDS sample buffer (Invitrogen), resolved by SDS-PAGE on 4 to 12% Novex bis-tris precast gels (Thermo Fisher Scientific), and analyzed by Western blotting using a wet transfer method (GenScript) and polyvinylidene difluoride membranes (Life Technologies). The membranes were probed with mouse anti-human CFTR monoclonal antibody no. 596 or no. 660 (1:2000; Cystic Fibrosis Foundation) targeting NBD2 and NBD1, respectively, or mouse anti-human CFTR MAB3482 (1:200; Millipore) targeting MSD1 and rabbit anti–glyceraldehyde phosphate dehydrogenase (GAPDH; 1:5000; #ab9485, Abcam) or rabbit anti-α/β-tubulin (1:2000l Cell Signaling Technology, #2148) or mouse anti-ubiquitin (1:500; Santa Cruz, #P4D1) or rabbit-anti-ubiquitin (1:200; Abcam, #ab140601). Secondary antibodies were sheep horseradish peroxidase (HRP)–coupled anti-mouse immunoglobulin G (IgG; Sigma-Aldrich, GENA931) and donkey HRP–coupled anti-rabbit IgG (Sigma-Aldrich, GENA934) were used at 1:5000 dilution. Membranes were treated with Western Lightning enhanced chemiluminescence substrate (PerkinElmer), and the chemiluminescence signal was recorded and quantified using a chemiluminescence reader and the corresponding software (Fusion FX 7, Evolution Edge, Vilber). For quantification, pixel intensities for CFTR were normalized against pixel intensities of loading controls (GAPDH or α/β tubulin).

### Immunofluorescence microscopy

CFBE-WT-CFTR and CFBE-F508del-CFTR cells were seeded at 10,000 cells per well onto FNC-coated eight-well chamber slides for a few hours and incubated with fixed concentrations of correctors or corresponding vehicle for 24 hours. Then, cells were washed once with PBS, fixed with 3% paraformaldehyde (37°C, 10 min) in PBS, washed twice with PBS, and then blocked with 10% FBS and 0.1% saponin in PBS (blocking buffer) for 1 hour at room temperature. Cells were then stained with mouse monoclonal anti-CFTR antibody 570 (1:200 in above blocking buffer; Cystic Fibrosis Foundation) and rabbit polyclonal anti–ZO-1 antibody (1:200; Thermo Fisher Scientific #40-2200) for 2 hours at room temperature and then washed three times in blocking buffer, followed by the addition of secondary antibodies in blocking buffer [goat-anti-rabbit-IgG-Alexa Fluor 488 (Thermo Fisher Scientific #A11034) and goat-anti-mouse-IgG-Alexa Fluor 568 (Thermo Fisher Scientific #A11031) both 1:200] and Hoechst 33342 (2 μg/ml from 10 mg/ml stock, Thermo Fisher Scientific #H3570). After 30 min at room temperature, cells were washed three times with blocking buffer and two times with PBS and mounted. Images were taken with fixed settings using the Leica confocal microscope TCS SP5 and the 63× objective. Z-stacks of 10 slices (0.9-μm distance) were taken and fused into one image by maximal intensity projection.

### Transepithelial short-circuit current measurements in reconstituted patient tissue

CFTR function was determined by the measurement of transepithelial short-circuit currents in reconstituted patient tissue using the using chamber. Tissues were either commercially available (MucilAir, Epithelix) or prepared in-house from hAECs (Epithelix, Lonza). Measured tissues originated from six F508del homozygous patients (CF patient #1: CF-AB0609, female, 31 years old; CF patient #3: #28388-0000450918, Lonza, male, 25 years old; CF patient #4: CF-MD0637 male, 22 years old; CF patient #6: CF-MD0519, female, 33 years old; CF patient #5: CF-MD0526, female, 16 years old; CF patient #7: CF-MD0567, female, 39 years old;), two F508del heterozygous patients (CF patient #8: CF-MD0638, F508del/2789 + 5G->A, male, 39 years old; CF patient #9: CF-MD0487, F508del/2183AA->G, male, 24 years old), and three non-CF donors (non-CF donor #5: WT-MD0810, female, 52 years old; non-CF donor #2: WT-AB0834, male, 71 years old; non-CF donor #4: CC-2540S-20TL256517, Lonza, female, 48 years old). After coincubation with correctors for 24 hours in medium (MucilAir medium, Epithelix) supplemented with 60% human serum (#4522, Sigma-Aldrich) at 37°C, 5% CO_2_, the inserts were mounted in Ussing chamber systems (EM-CSYS-8, Physiologic Instruments, San Diego CA, USA). The basal buffer was composed of 110 mM NaCl, 1.2 mM CaCl_2_, 1.2 mM MgCl_2_, 2.4 mM Na_2_HPO_4_, 0.4 mM NaH_2_PO_4_, 25 mM NaHCO_3_, 5.2 mM KCl, and NaOH to adjust the pH to 7.4, while the apical buffer contained 120 mM Na-gluconate, 1.2 mM CaCl_2_, 1.2 mM MgCl_2_, 2.4 mM Na_2_HPO_4_, 0.4 mM NaH_2_PO_4_, 25 mM NaHCO_3_, 5.2 mM KCl, and NaOH to adjust the pH to 7.4, the basal-to-apical chloride gradient facilitating the recording of chloride currents. During experiments, both buffers were maintained at 37°C and continuously gassed with a O_2_-CO_2_ gas mixture (95 to 5%). Ag/AgCl electrodes filled with 3 M KCl were connected to both hemi-chambers to VCC MC8 voltage/current clamp amplifiers (Physiologic Instruments). Transepithelial short-circuit currents were recorded at 1-Hz sampling frequency using the Acquire and Analyze software version 2.3.8 (Physiologic Instruments) during a baseline period and upon sequential addition of 100 μM amiloride (Sigma-Aldrich), 5 μM forskolin (Sigma-Aldrich), 1 μM IVA, 100 μM uridine triphosphate (Sigma-Aldrich), and 20 μM Inh172 (Sigma-Aldrich). Offline analysis of short-circuit currents was performed with Excel and Prism 8 (GraphPad, San Diego, CA, USA) and then normalized against the surface of the inserts.

### Halide-sensitive YFP quenching assay

U2OS-F508del-CFTR cells expressing Topaz-YFP F46L/H148Q/I152L were seeded at 20,000 cells per well into 384-well black clear-bottom plates in 40 μl per well growth medium (Mc COY’s 5A, Gibco, 10% FBS, penicillin/streptomycin), containing the various CFTR correctors at the indicated concentrations. The cells were coincubated with the compounds for 24 hours (37°C, 5% CO_2_). The next day, plates were washed twice with PBS^+^ (55 μl per well; PBS containing 0.9 mM Ca^2+^ and 0.5 mM Mg^2+^). PBS^+^ was fully removed, and cells were supplemented with 15 μl PBS^+^. Then, 5 μl of 4× concentrated stocks of IVA or vehicle in dilution buffer [PBS^+^, 0.1 μM forskolin, and 0.2% bovine serum albumin (fatty acid free) (pH 7.4)] were added and incubated for 30 min in the dark. For experiments with I-172 (Sigma- Aldrich), the inhibitor was added to the mix with either IVA or vehicle to reach a final concentration in cells of 20 μM. Then, plates were transferred to the FLIPR Tetra (fluorescence imaging plate reader; Molecular Devices: excitation: 470 to 495 nm; emission: 526 to 585 nm), baseline fluorescence reading of the YFP signal was performed for 6 s (10 × 0.6 s intervals) after which 25 μl of iodide buffer [137 mM NaI, 2.7 mM KCl, 1.5 mM KH_2_PO_4_, 8.1 mM Na_2_HPO_4_, 1 mM CaCl_2,_ and 0.5 mM MgCl_2_ (pH 7.4)] was added, and fluorescence reading was continued for 70 s (50 × 0.6 s intervals; 20 x 2 s intervals) to assess YFP quenching through CFTR-mediated iodide influx. For analysis, fluorescence traces were aligned and normalized at the last time point before iodide addition (normalized fluorescence = 1). Normalized fluorescence values obtained 20 s after the beginning of the assay were used to assess the degree of YFP quenching, i.e., CFTR function (see fig. S1J). For copotentiation experiments, HEK-G551D-CFTR were seeded at 40,000 cells per well into 384-well black clear-bottom plates coated with 0.01% poly-l-lysine [Sigma-Aldrich, #P8920, 0.1% (w/v) diluted 1:10 in PBS−] in HEK growth medium (40 μl per well). Cells were induced with tetracycline (10 ng/ml) for 24 hours (37°C, 5% CO_2_). The next day, plates were washed twice with PBS^+^ (55 μl per well). PBS^+^ was fully removed, and cells were supplemented with 15 μl of PBS^+^. IVA in different concentrations or vehicle was mixed together with vehicle or correctors at the indicated concentrations (acute treatment). Then, 5 μl of 4× concentrated stocks of compound mix in dilution buffer [PBS^+^, 0.1 μM forskolin, and 0.2% bovine serum albumin (fatty acid free) (pH 7.4)] was added and incubated for 10 min in the dark. Then, plates were read and analyzed as above. For analysis of WT-CFTR, CFBE41o^−^ cells expressing WT-CFTR and Topaz-YFP F46L/H148Q/I152L were seeded at 20,000 cells per well into 384-well black clear-bottom plates coated with FNC coating mix (Enzo, #0407) in CFBE growth medium (40 μl per well). Cells were induced with vehicle or 2 μM IDOR-4 for 24 hours (37°C, 5% CO_2_). The next day, plates were washed twice with PBS^+^ (55 μl per well). PBS^+^ was fully removed, and cells were supplemented with 15 μl of PBS^+^. Then, 5 μl of 4× concentrated IVA in dilution buffer [PBS^+^ and 0.2% bovine serum albumin (fatty acid free) (pH 7.4)] was added and incubated for 30 min in the dark. Then, plates were transferred to the FLIPR Tetra and read and analyzed as above.

### Metabolic pulse-chase and chase assays to determine CFTR folding efficiency and half-life

Stable isotope labeling using amino acids in cell culture (SILAC technology, Thermo Fisher Scientific) was used. For pulse-chase assays to determine CFTR folding efficiency at the ER, CFBE-F508del-CFTR or CFBE-WT-CFTR were seeded at 100,000 cells per well into a FNC-coated 24-well plate in CFBE growth medium and, the next day, incubated for 24 hours with “SILAC light medium” [arginine- and lysine-free RPMI 1640 #A33823, 84 mg/liter light L-arginine, # 89989, 146 mg/liter light L-lysine, #88429 and 20 mg/liter L-proline, # 89989 (to prevent a potential arginine-proline conversion)] supplemented with 10% dialyzed FBS. Cells were then washed once in PBS (37°C) and incubated for 1 hour with “starvation medium” (arginine-, lysine-free RPMI 1640, and 1% HSA) containing the indicated fixed concentrations of correctors. A 30-min metabolic pulse labeling was then performed by replacing the medium with “SILAC heavy medium” [arginine- and lysine-free RPMI 1640, l-proline (20 mg/liter), heavy l-arginine (84 mg/liter; ^13^C_6_^15^N_4_, #89990), heavy l-lysine (146 mg/liter; ^13^C_6_^15^N_2_, #88209), and 1% HSA] containing correctors. Cells were then washed with PBS and chased in SILAC light medium supplemented with 1% HSA and correctors. This medium was kept for 1, 2.5, 4, and 6 h of chase time followed by cell lysis in RIPA buffer as described above. For chase assays to determine CFTR C-band half-life, CFBE-F508del-CFTR or CFBE-WT-CFTR were seeded as above and incubated for 24 hours with SILAC light medium supplemented with 1% HSA and the indicated concentrations of correctors. Cells were then washed once in PBS (37°C) and incubated for 1 hour with starvation medium, followed by incubation with SILAC heavy medium both containing the correctors for 0.5, 1, 2.5, 4, 6, 8 to 9, and 24 hours of chase times followed by cell lysis. Cells for wash-out analysis, before addition of SILAC heavy medium, were washed twice in CFBE growth medium and once in PBS and then incubated for the indicated chase times in SILAC heavy medium without correctors. For the chase assay in primary ALI tissues, fully differentiated tissues from CF patient #2 and non-CF donors #2 and #3 were used, and the experiment design was the same as for CFBE cells with all SILAC media supplemented with 1% HSA and indicated correctors added directly at the basal side of ALI tissues. Samples were chased for 1, 2.5, 6, 8, and 25 hours, followed by lysis in RIPA buffer as described above.

### Cross-linking with photo-activatable probes

U2OS-F508del-CFTR cells were seeded at 120,000 cells per well in a 24-well plate for a day and then incubated for 48 hours with 1 to 2 μM each probe (photo-activatable IDOR-5A, IDOR-6A, and IDOR-76B; non-photo-activatable controls IDOR-5 and IDOR-6) diluted in the growth medium. Medium was then removed, and the ice-cooled cell plate was UV-irradiated (254 nm) at close distance (2 cm) without lid for 5 min (7 W, Camag). Cells were then lysed in RIPA buffer as described above. Control plates were not exposed and directly lysed.

### Mass spectrometry–based quantification of peptides

Cell lysates from pulse-chase, chase assays, or cross-linking experiments were processed for LC-MS analysis. Samples were mixed with LDS sample buffer, resolved by SDS-PAGE on 3 to 8% tris-acetate precast gels (Thermo Fisher Scientific), followed by protein staining with InstantBlue (Bio-Techne AG). CFTR protein bands were excised, cut in small pieces (1 to 2 mm^3^), moved to a Protein LoBind 96-well plate (Eppendorf), and destained with several alternating washes of freshly prepared solutions of 50 mM ammonium bicarbonate (Ambic) and 50 mM Ambic in 50% acetonitrile (ACN), until the color was completely removed. Gel pieces were then dried under nitrogen and incubated for up to 1 hour with a 5 mM Tris(2-carboxyethyl)phosphin-50 mM iodoacetamide in 50 mM Ambic solution for protein reduction and alkylation. After washes with 50 mM Ambic in 50% ACN, gel pieces were dried again and incubated with a freshly prepared solution trypsin (12.5 ng/μl; sequence grade, Promega) in 50 mM Ambic for 16 to 20 hours at 37°C. Peptides were extracted from gels with one volume of 50 mM Ambic and two volumes of 5% formic acid (FA) in 50% ACN and collected in a new plate. Samples were concentrated to one/third of their volume and resuspended in 0.1% trifluoracetic acid (TFA) in H_2_O. Peptide solutions were loaded onto OASIS μElution 96-well plates (Waters), for desalting and enrichment. After several washes in 0.1% TFA, peptides were eluted under vacuum with 60 μl of 70% ACN in 0.1% TFA, collected in a new LoBind plate, and dried and resuspended in 30 μl of a 2% ACN and 0.1% TFA solution.

The peptides were quantified on a Thermo Scientific Orbitrap Exactive HF-X instrument [Thermo Fisher Scientific connected to an UltiMate 3000 RSLC nano system (Thermo Fisher Scientific)]. The data were analyzed using Rmarkdown with R version 4.1.1 (2021-08-10), and plots were generated using the ggplot2 R package.

For metabolic pulse-chase and chase assays, typically, 10 μl of the peptide solution was injected and quantified using a 30-min method. Peptides were loaded first on a Trap column (Acclaim PepMap 100 3-μm C18 column of 2-cm length and 75-μm inner diameter, Thermo Fisher Scientific) and then injected on an analytical column (EasySpray 15-cm column 2-μm 100-Å C18 column with 75-μm inner diameter, Thermo Fisher Scientific) and separated using a 10-min gradient from 5% buffer B (80% ACN and 0.1% FA in H_2_O) in buffer A (0.1% FA in H_2_O) to 35% buffer B. The eluting peptides were acquired using a MS1 spectra [200 to 1200 mass/charge ratio (*m/z*), resolution of 60,000, automatic gain control target of 3 × 10^6^, and maximum injection time of 120 ms] followed by up to 39 MS2 spectra (isolation window 1.4 *m/z* with 0.2 *m/z* offset, 45,000 resolution, automatic gain control target of 2 × 10^5^, and maximum injection time of 86 ms) using a scheduled injection list for CFTR peptides, peptides of proteins for QC assessment of the gel cutting, and three iRT peptides (Biognosys, Schlieren).

Data analysis of the signal acquired in parallel acquisition mode was performed using Skyline [MacCoss Lab, University of Washington (*41*)]. The signal for four to six fragments for each peptide were integrated by automatic peak picking and manually controlled. The data were then exported to tab-delimited file for analysis using an in-house developed Rmarkdown script analyzing the total signal for all fragments for the eight peptides (AVQPLLLGR, GQLLAVAGSTGAGK, NSILNPINSIR, LSLVPDSEQGEAILPR, ISVISTGPTLQAR, STLLSAFLR, VFIFSGTFR, and VADEVGLR) from the CFTR protein. As a QC step, we also analyzed QC peptides identified to comigrate with the B- and C-bands of CFTR [C-band: CLTC (theoretical molecular weight of protein sequence, 192 kDa) (VANVELYYR and HELIEFR) and SMC4 (147 kDa) (VLDAIIQEK and LLEENVSAFK); B-band: MED23 (156 kDa) (LFDLLYPEK and YNIVTLDR) and LRPPRC (158 kDa) (SSLLLGFR and IPENIYR) to confirm the precision of gel excision and separation of CFTR B- and C-bands] (see fig. S3C).

Different peptides at the same concentration will give a different signal based on how easy they ionize and fragment, i.e., their response factor (see, for an example, fig. S3A). In this study, a relative response factor was calculated per experiment in the WT-CFTR cells for the light isotopolog relative to the AVQPLLGR peptide (see fig. S3B for an example). The signal of each peptide from CFTR was then normalized by their relative response factor. For the pulse-chase experiments, this normalized intensity was then further normalized to the maximal intensity observed in the B-band for the heavy isotopolog at time point 0. See table S1 for peptide analysis details.

For the cross-linking experiments, 10 μl of the gel-extracted peptide solution was injected and quantified using a 140-min method. Peptides were loaded first on a Trap column (Acclaim PepMap 100 3-μm C18 column of 2-cm length and 75-μm inner diameter, Thermo Fisher Scientific) and then injected on an analytical column (EasySpray 50-cm column 2-μm 100-Å C18 column with 75-μm inner diameter, Thermo Fisher Scientific) and separated using a 114-min gradient from 2% buffer B (80% ACN and 0.1% FA in H_2_O) in buffer A (0.1% FA in H_2_O) to 35% buffer B. Masses from eluting peptides were acquired using a MS1 spectra (400 to 1200 *m/z*, resolution of 120,000, automatic gain control target of 3 × 10^6^, and maximum injection time of 20 ms) followed by up by 40 MS2 spectra in data-independent acquisition (DIA) mode from 400 to 1000 *m/z* (isolation window of 16 *m/z* with 100 *m/z* first mass, 30,000 resolution, automatic gain control target of 5 × 10^5^, maximum injection time of 55 ms, and normalized collision energy of 27). The data were then analyzed using a directDIA workflow in Spectronaut version 15 (Biognosys) using standard settings and a fasta file containing the mutated F508del-CFTR protein sequence. Transition-level data were exported and further filtered using in-house R scripts and mapDIA (*42*), and the intensities of three to five fragments per peptide are summed for analysis. The signal of each peptide was normalized to the total CFTR signal (summed signal of all CFTR peptides per sample) and to the average signal across all conditions of that respective peptide to account for the different response factors. Protein coverage in fig. S5E was displayed using the Protter (*43*).

### Chromatographic determination of probes concentrations upon UV light exposures

Stock solutions of IDOR-5A, IDOR-6A, IDOR-6B, IDOR-6, and IDOR-3 were prepared at 10 mM in DMSO. A series of 12 working standard solutions for calibration curve were prepared at concentrations from 250 to 1.22 nM by twofold serial dilution. A total of 2 μM dilutions of the same compounds in 50 mM tris buffer, exposed or not for 5 min to UV light as above, were diluted 10-fold with H_2_O/ACN 50/50 v. Samples were then mixed and centrifuged at 3700*g* for 15 min prior injection. The measurements were performed on a high-performance liquid chromatography (HPLC) Nexera X2 HPLC (Shimadzu) coupled to an API 5500TM (AB Sciex) with positive ion electrospray ionization and operated in multiple reaction monitoring (MRM) mode. Data were collected and processed by Analyst 1.6.2, and the chromatographic separation was carried out on a Acquity HSS T3 1.8 mm, 2.1 × 50 mm (Waters Corp) at 40°C. The separation method consisted in a gradient elution of the mobile phase (0.1% FA in A: water and B: ACN) was as follows: 100% B at 0 min and 98% B at 1.5 min and held for 0.6 min before re-equilibration. Total run time was 2.5 min, and all samples were analyzed with an injection of 1 ml. The source parameters were curtain gas nitrogen: 20 psig; collision gas: 6 psig; ion source gas 1: 20.0 psig; ion source gas 2: 20.0 psig; ion spray voltage: 4500 V; turbo heater temperature: 500°C; entrance potential: 10 V. The electrospray ionization was operated in positive MRM mode after optimization according to the standard procedure. Compound-specific values of mass spectrometer parameters are listed in table below.

### CFTR mRNA quantification

CFBE-F508del-CFTR cells were seeded at 70,000 cells per well in a FNC-coated 48-well plate and incubated for 24 hours with 2000 nM of indicated correctors or DMSO vehicle diluted in growth medium deprived of FBS but supplemented with 1% HSA. CFBE-WT-CFTR cells were treated with DMSO vehicle accordingly. Then, cells were washed twice with PBS^+^ and lysed in RLT buffer (QIAGEN) for 10 min on ice. Lysates were fully homogenized with QIAshredder (#79656, QIAGEN), and total RNA was extracted with an RNeasy Mini kit (#74106, QIAGEN) following the manufacturer’s instruction. At the end, a deoxyribonuclease treatment was performed with a DNA-free kit (Invitrogen, AM1906) following the manufacturer’s instruction. A reverse transcription (150 ng of total RNA per sample) with random hexamers was performed with the high-capacity cDNA Reverse Transcription Kit (#4368814, Thermo Fisher Scientific) for 2 hours at 37°C. Three microliters of cDNA was mixed with 10 μl of TaqMan Fast Universal PCR (polymerase chain reaction) Master Mix (2×) No AmpErase UNG (4367848, Life Technologies) containing the probe of interest (1:20), and a quantitative PCR was run [20″ at 95°C; 3″ at 95°C to 30″ at 60°C (40 cycles)] on a 7500 Applied Biosystems device. TaqMan (FAM-MGB) probes: *CFTR* (Hs00357011_m1) was used to detect the target gene; 18*S* (Hs99999901_s1), *GUSB* (Hs00939627_m1), and *PPIA* (Hs04194521_s1) were used as reference genes for normalization. Reference gene-normalized CFTR mRNA expression was calculated using ΔΔCt method with CFTR expression in vehicle-treated CFBE-F508del cells set to 1.

### Three-dimensional modeling

All computational calculations were performed with the phosphorylated structure of human CFTR [Protein Data Bank (PDB): 6MSM] and F508del-CFTR (PDB: 8EIQ). The two Mg-ATP complexes were kept, and the membrane was positioned using the Orientations of Proteins in Membranes (OPM) database (*44*). F508 was deleted and the immediate surrounding was subject to minimization using Maestro (https://schrodinger.com/products/maestro). Docking and Molecular Dynamic Simulations were conducted with Glide XP (*45*) and Desmond (*46*) (Schroedinger suite), respectively. Molecular dynamics simulations used F508del-CFTR embedded in a 1-palmitoyl-2-oleoyl-glycero-3-phosphocholine lipid bilayer. The overall system was solvated (TIP3P) and neutralized with 150 nM NaCl. Each simulation (250 ns) was conducted at 300 K using the default parameters.

### Statistics

For evaluation of corrector efficacies on second-site suppressor mutants in HEK cells, a one-way analysis of variance (ANOVA) test with Turkey’s multiple comparisons posttest was done between the three different mutants within each compound treatment. The same test was performed to evaluate corrector efficacies in binding sites double or triple mutants, between the four different corrector treatments within every mutant. Statistical tests were done with GraphPad Prism 8.

To identify peptides that showed lower signal upon cross-linking, the normalized value for all >80 quantified peptides of CFTR was statistically compared across conditions using pair-wise testing for a loss of signal (i.e., one-sided *t* test), using R software 4.1.1. We compared the UV-excited conditions of samples treated with each compound to their respective non-exposed controls. The signal of each cross-linkable compound after UV excitation was also compared to the respective UV-excited noncross-linkable parent compound (IDOR-5A versus IDOR-5 and IDOR-6A or IDOR-6B versus IDOR-6). To control for multiple testing of the about 80 peptides in each comparison, a multiple testing correction using Benjamini-Hochberg was performed (*P*_adj_). With these stringent statistical criteria, only two peptides, LFFSWTR and LFFSWTRPILR, showed a statistically significant difference in signal after multiple testing correction (*P*_adj_ = 2.0·10^−4^ and *P*_adj_ = 1.2·10^−5^, respectively) in the comparison of IDOR-6A versus IDOR-6. No other peptide in no other tested comparison showed an adjusted *P* value below 0.05 (fig. S5F). The results for these two peptides without multiple testing correction showed that there was also a statistical different signal between IDOR-6A–treated samples after and without UV excitation (*P* = 7.5·10^−4^ and *P* = 1.4·10^−3^, respectively) but not in any other comparison.
