# Supplementary material for: A uniquely efficacious type of CFTR corrector with complementary mode of action
Source: Sci Adv. 2024 Mar 1;10(9):eadk1814. doi: 10.1126/sciadv.adk1814 (PMC11801377; doi:10.1126/sciadv.adk1814)
Supplement: Supplementary file 1 — Figs. S1 to S7 Tables S1 and S2 [file sciadv.adk1814_sm.pdf]

Science Advances
AAAS

Supplementary Materials for

A uniquely efficacious type of CFTR corrector with complementary mode of action

Valentina Marchesin *et al.*

Corresponding author: John Gatfield, john.gatfield@idorsia.com

Sci. Adv. **10**, eadk1814 (2024)
DOI: 10.1126/sciadv.adk1814

This PDF file includes:

Figs. S1 to S7
Tables S1 and S2

SUPPLEMENTAL FIGURES

A

	Assay	Compounds	Cut-off	Resulting compounds
Primary screen at 10μM	U2OS-F508del-CFTR	67772	Activity > 13% vs 3μM LUM	129 (0.19%)
Hit confirmation at 10μM in duplicates	U2OS-F508del-CFTR	129	Activity > 13% vs 3μM LUM	101 (0.15%)
Counter-screening at 10μM in duplicates	U2OS-ADRB2-W158A	101	Activity < 30% vs 100nM propanolol	83 (0.12%)
EC50 / Emax determination	U2OS-F508del-CFTR	83	Emax > 50% activity vs Emax LUM<br>EC50 < 2μM	4

B

Corrector Type		EC_{50} (nM)	E_{max} (fold baseline)
I	TEZ	320	4.6
	LUM	200	4.7
	GAL	14	4.4
II	C4a	2400	4.4
III	ELX	303	6.8
	BAM	218	8.7
I+III	TEZ+ELX	250	9.8
IDOR	IDOR-0	1600	3.0
	IDOR-1	564	7.0
	IDOR-2	108	14
	IDOR-3	158	15
	IDOR-4	18	18

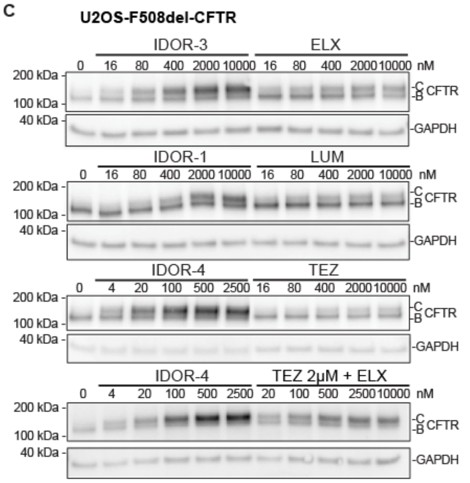

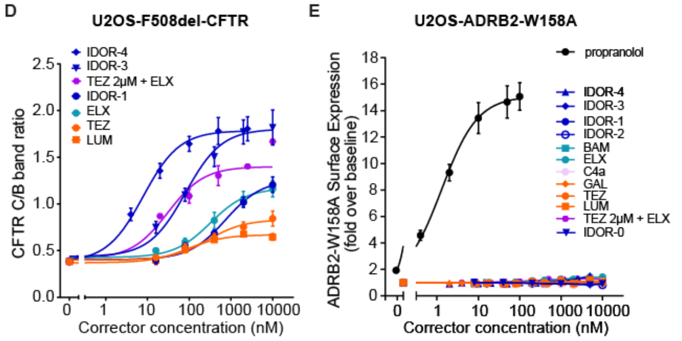

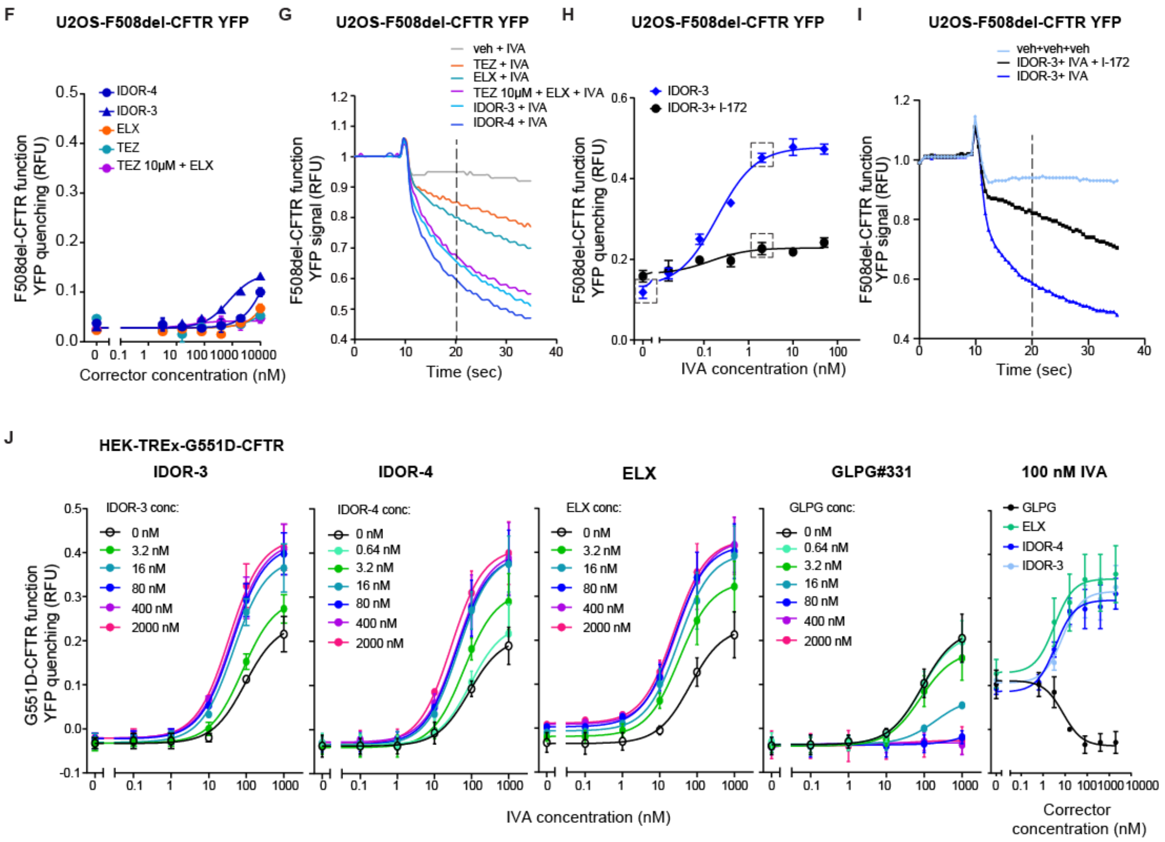

Fig. S1. Related to Fig. 1: Type-IV correctors restore F508del-CFTR trafficking to the cell surface and behave additively with type-I, type-II, and type-III correctors. (A) *High throughput screening statistics indicating the cell lines used for screen and counter-screen, the screening concentrations, the activity cut-offs vs references (LUM and propranolol) and the resulting hits and hit rates. 3μM LUM and 100 nM propranolol served as 100% activity controls in*

the respective assays. Four confirmed hits were specific (vs ADRB2-W158A), potent (EC_{50} >2μM) and efficacious (E_{max}>50%) one of which was macrocycle IDOR-0. ***(B)*** *EC_{50} and efficacious E_{max} values deduced from the corrector concentration response curves shown in Figure 1A.* ***(C)*** *Immunoblot analysis of CFTR and GAPDH expression in U2OS-F508del-CFTR cells treated over-night with different concentrations of the indicated correctors. Representative images of n=7 independent experiments.* ***(D)*** *Quantification of the CFTR C-band intensity normalized for the B-band (n=2 for TEZ+ELX combination, n=7 for all other compounds).* ***(E)*** *ADBR2^{W158A} surface expression in U2OS cells upon over-night treatment with dilution series of the indicated correctors or adrenergic receptor ligand propranolol (n=3 for TEZ+ELX combination, n=5 for all other compounds).* ***(F)-*** *F508del-CFTR function (Topaz-YFP$^{F46L.H148Q.I152L}$quenching assay) after 24 h treatment of U2OS-F508del-CFTR-YFP cells with a concentration response of IDOR-3, IDOR-4, TEZ, ELX or TEX(10μM)+ELX in the acute presence of 0.1 μM forskolin (n=2);* ***(G)*** *Examples of raw YFP quenching traces of one of the experiments shown in Fig 1C (veh or 10000 nM of IDOR-3 or IDOR-4 or ELX or TEZ or TEZ+ELX). Fluorescence values obtained 20 seconds from the beginning of the measurement were used to assess the degree of YFP quenching.* ***(H)*** *Effect of 2000 nM of IDOR-3 in presence or absence of different concentrations of IVA and with or without 20 μM of CFTR-inhibitor-172 (n=3). Enclosed in dashed squares are the conditions shown in (I).* ***(I)*** *Examples of raw YFP quenching traces of one of the experiments shown in I (veh or 2000 nM IDOR-3 + 2 nM IVA or 2000 nM IDOR-3 + 2 nM IVA + 20 μM I-172). Fluorescence values obtained 20 seconds from the beginning of the measurement were used to assess the degree of YFP quenching.* ***(J)*** *G551D-CFTR function (YFP quenching assay) in HEK-TREx-G551D-CFTR-YFP cells exposed to acute treatment (10 min) with different concentrations of IVA combined with different concentrations of IDOR-3, IDOR-4, ELX or GLPG #331 (n=2). The right panel replots the data as corrector concentration-response curves for the 100 nM IVA condition. Data in (D), (E), (F), (H) and (J) are means ± SEM of the indicated number of independent experiments.*

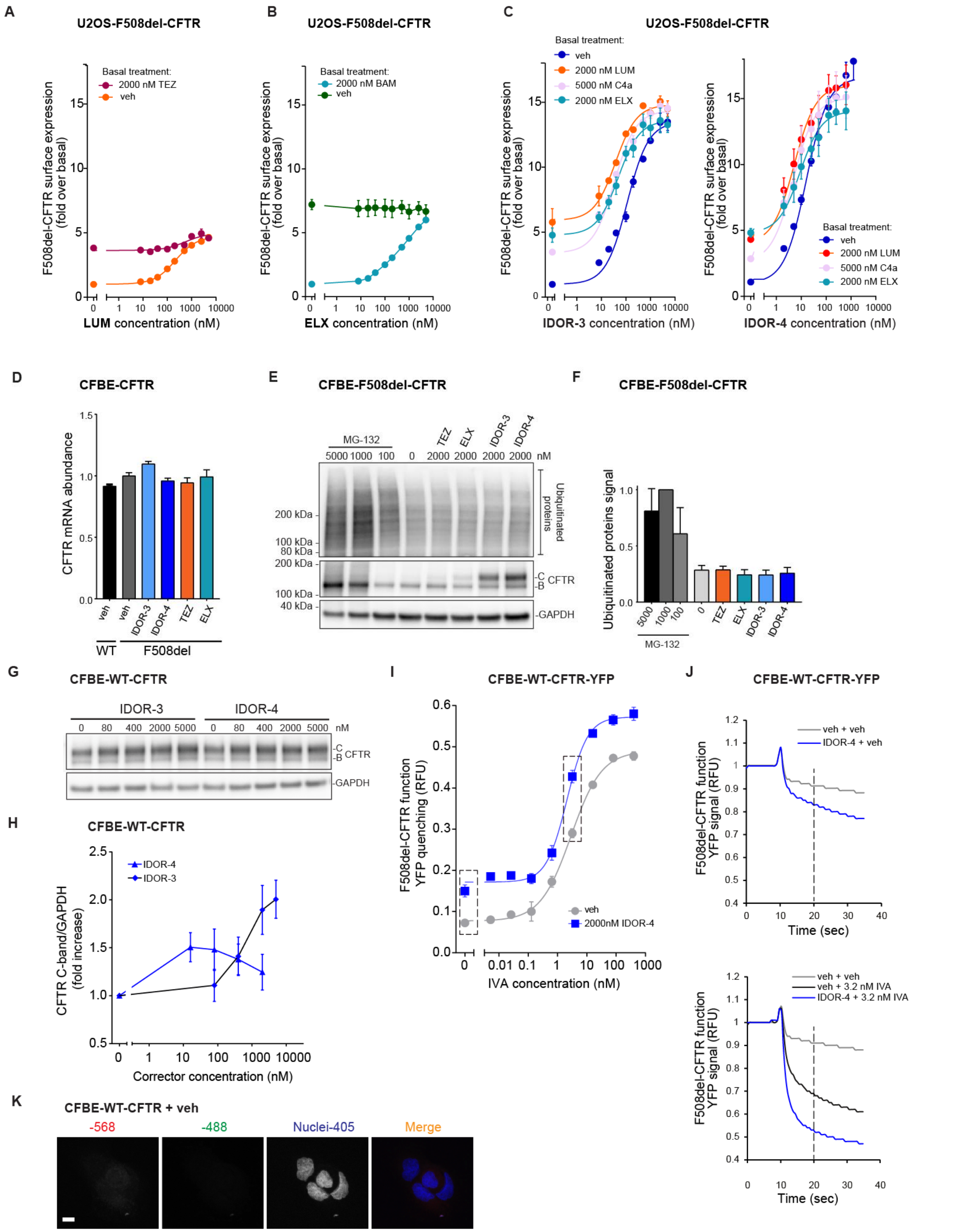

Fig. S2. Related to Fig. 1: Type-IV correctors restore F508del-CFTR trafficking to the cell surface and behave additively with type-I, type-II, and type-III correctors. (A-C) *CFTR surface expression in U2OS-F508del-CFTR cells after over-night treatment with: A) different concentrations of LUM in presence of vehicle or a maximally effective concentration of TEZ (n=3). B) different concentrations of ELX in presence of vehicle or a maximally effective concentration of BAM (n=4). C) different concentrations of IDOR-3 or IDOR-4 in presence of vehicle or a maximally effective concentration of the indicated other correctors (n=3);* ***(D)*** *Quantification of CFTR mRNA expression in CFBE41o- cells expressing WT-CFTR (n=2) or F508del-CFTR after 24 h of treatment with DMSO (n=3) or 2000 nM of*

the indicated correctors (n=2). ***(E)*** *Immunoblot analysis of levels of ubiquitinated proteins, CFTR and GAPDH in CFBE-F508del-CFTR cells after 24 h of treatment with DMSO, MG-132 or the indicated correctors. Representative images (n=3).* ***(F)*** *Quantification of ubiquitinated proteins in (E), normalized for GAPDH (n=3).* ***(G)*** *Immunoblot analysis of WT-CFTR-expressing CFBE41o- cells treated for 24 h with different concentrations of the indicated correctors and probed for CFTR and GAPDH. Representative images (n=3).* ***(H)*** *Quantification of the CFTR C-band intensities in (G) normalized for GAPDH (n=3).* ***(I)*** *YFP-quenching assay: Analysis of CFTR function in CFBE41o- cells expressing haldide-sensitive YPF and WT-CFTR were treated over night with vehicle or 2000 nM IDOR-4 and then stimulated with different IVA concentrations (n=2).* ***(J)*** *Representative raw traces for the boxed conditions in (I), namely 0 nM IVA and 3.2 nM IVA. Fluorescence values obtained 20 seconds from the beginning of the measurement were used to assess the degree of YFP quenching* ***(K)*** *Representative control immunofluorescence pictures for Figure 1G showing CFBE-WT-CFTR cells treated with DMSO for 24 h and stained for cell nuclei in blue and only secondary antibodies (red and green channels). Scale bar: 10μm. Data in (A), (B), (C), (D), (F), (H) and (I) are mean ± SEM of the indicated number of independent experiments.*

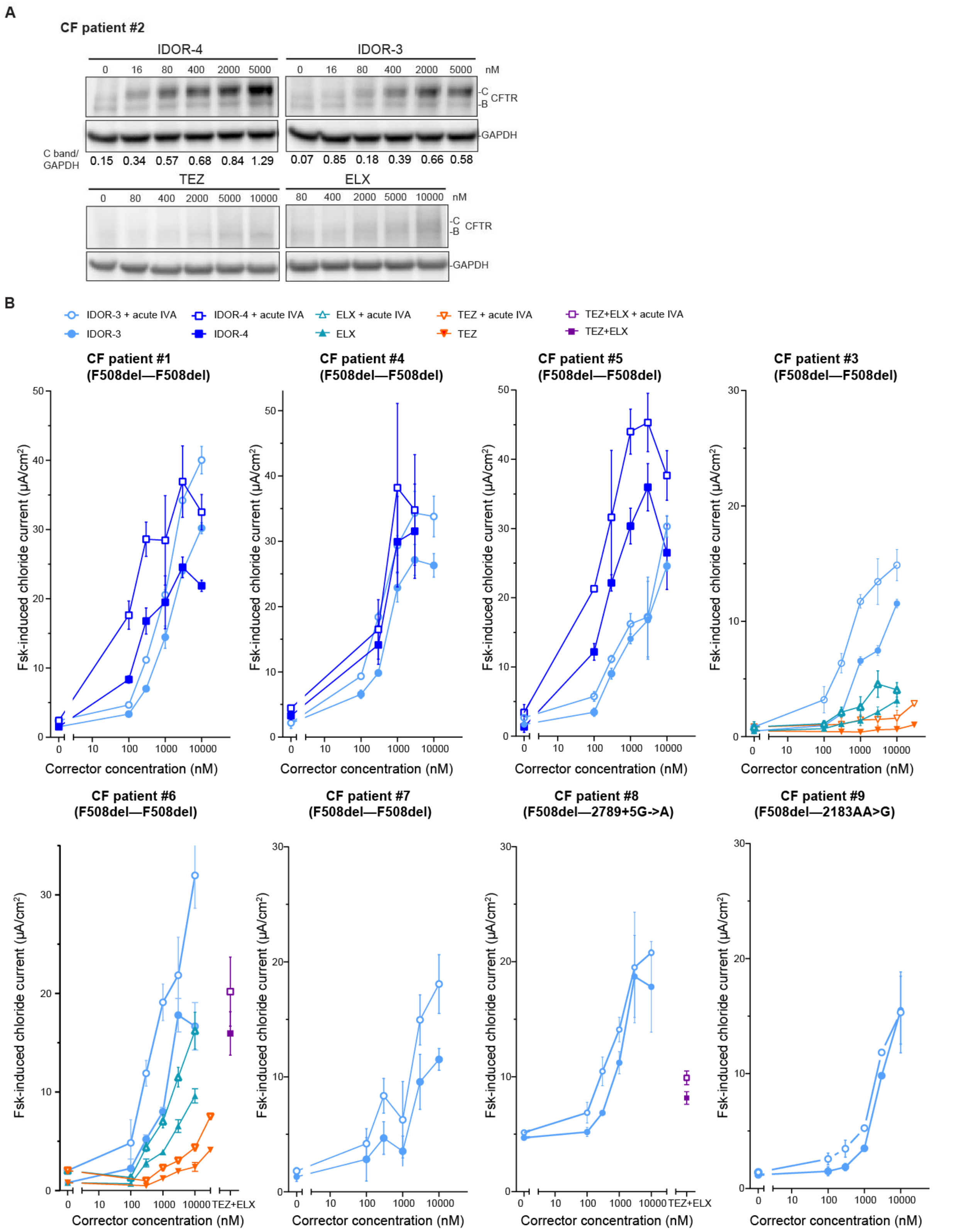

Fig. S3. Related to Fig. 2: Type-IV correctors rescue F508del-CFTR trafficking and function in reconstituted cystic fibrosis bronchial epithelium. (A) *CFTR expression (immunoblotting) in reconstituted human bronchial epithelium of CF patient #2 (F508del-CFTR homozygous) after a 24-h treatment with the indicated correctors. Representative images (n=2). The CFTR C-band/GAPDH intensity ratio is represented below every lane (for TEZ and ELX correction the ratio could not be calculated because the signal was too weak).* ***(B)*** *Measurement of transepithelial short-circuit currents in Ussing chamber in reconstituted epithelium of 8 CF patients (6 homozygous and two*

heterozygous for F508del-CFTR), treated over-night with vehicle or IDOR-3, IDOR-4, TEZ or ELX dilution series or the combination TEZ (15μM)+ELX (10μM), in presence or absence of acute 1 μM IVA (at least 2 independent measurements/patient). Data in (B) are mean ± SEM of the indicated number of independent experiments.

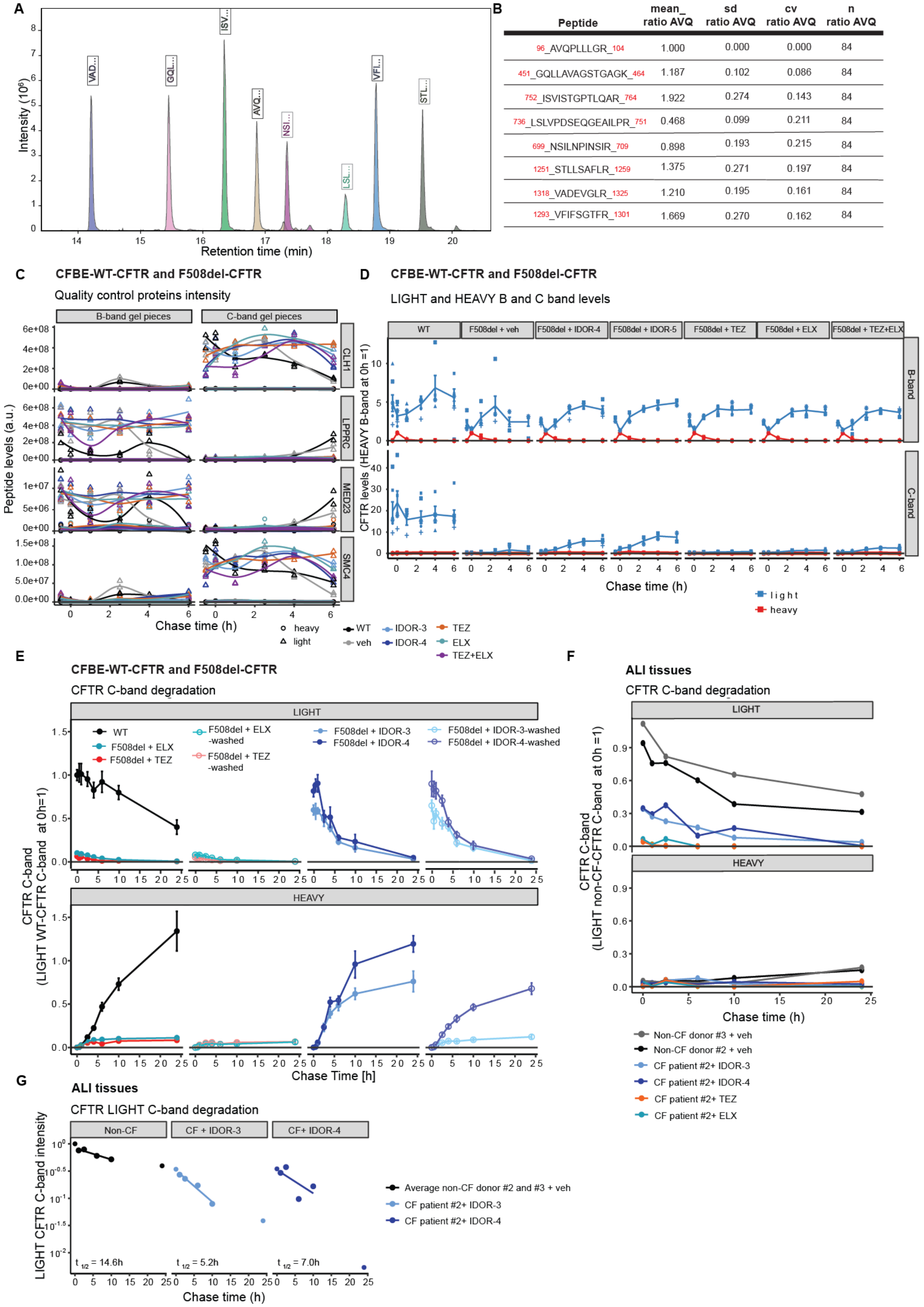

Fig. S4. Related to Fig. 3: Type-IV correctors restore F508del-CFTR folding efficiency in the endoplasmic reticulum beyond wildtype levels. (A) *Representative chromatogram showing the elution of the 8 peptides quantified for CFTR by mass spectrometry.* ***(B)*** *Representative normalization ratio of each peptide. All peptides are normalized to*

each other to account for the different response factor using the mean_ratio_AVQ which is the mean ratio of intensity between the respective peptide and the peptide AVQPLLLGR. ***(C)*** *Average peptide intensities of heavy-labeled (circles) and light-labelled (triangles) peptides of proteins predicted to co-migrate with CFTR-B band (LPPRC and MED23) or C-band (CLH1 and SMC4). Samples originate from pulse-chase experiments with CFBE cells expressing WT-CFTR or F508del-CFTR, pulse-labeled for 30 min with heavy amino acids and chased with light amino acids for the indicated time periods. During the whole assay F508del-CFTR cells were treated with DMSO (n=5) or 2 µM IDOR-3 (n=4), 2 µM IDOR-4 (n=2), 10 µM TEX or 10 µM ELX (n=3), indicated with different colors.* ***(D)*** *Average peptide intensities of heavy-labeled (red lines) and light-labeled (blue lines) CFTR B- (upper graphs) and C- (lower graphs) bands in CFBE cells treated as described under C). Peptide intensity per time point in each treatment is normalized to maximal intensity of heavy-labeled-B band at 0h chase time.* ***(E)*** *Average peptide intensities of light and heavy CFTR C-band in cells incubated for 24 h with light amino acids in the presence of DMSO (n=4) or 2 µM IDOR-3 or 1 µM IDOR-4 or 10 µM TEZ or 10 µM ELX (n=2 each) and then chased with heavy amino acids. Correctors were either kept during the chase period or thoroughly washed-out before the chase. Per experiment, peptide intensities at the different time points were normalized to the intensity of light C-band of CFTR WT at the beginning of the chase.* ***(F)*** *Peptide intensities of light- and heavy-labeled CFTR C-band in ALI tissues from non-CF donors #2 and #3 and CF patient #2, incubated for 24 h with light amino acids and DMSO or 2 µM IDOR-3, 1 µM IDOR-4, 10 µM TEZ or 10 µM ELX and then chased for the indicated time points with heavy amino acids. Per experiment, peptide intensities were normalized to the maximal intensity of the light-labeled C-band of WT-CFTR at the beginning of the chase (n=1).* ***(G)*** *Calculation of the light-labeled CFTR C-band half-life in ALI tissues from non-CF donors #2 and #3 and CF patient #2 corrected with IDOR-3 and IDOR-4, determined by linear regression of the logarithmic data using time points up to 10 h (n=1). Data in (C), (D) and (E) are mean ± SEM of the indicated number of experiments.*

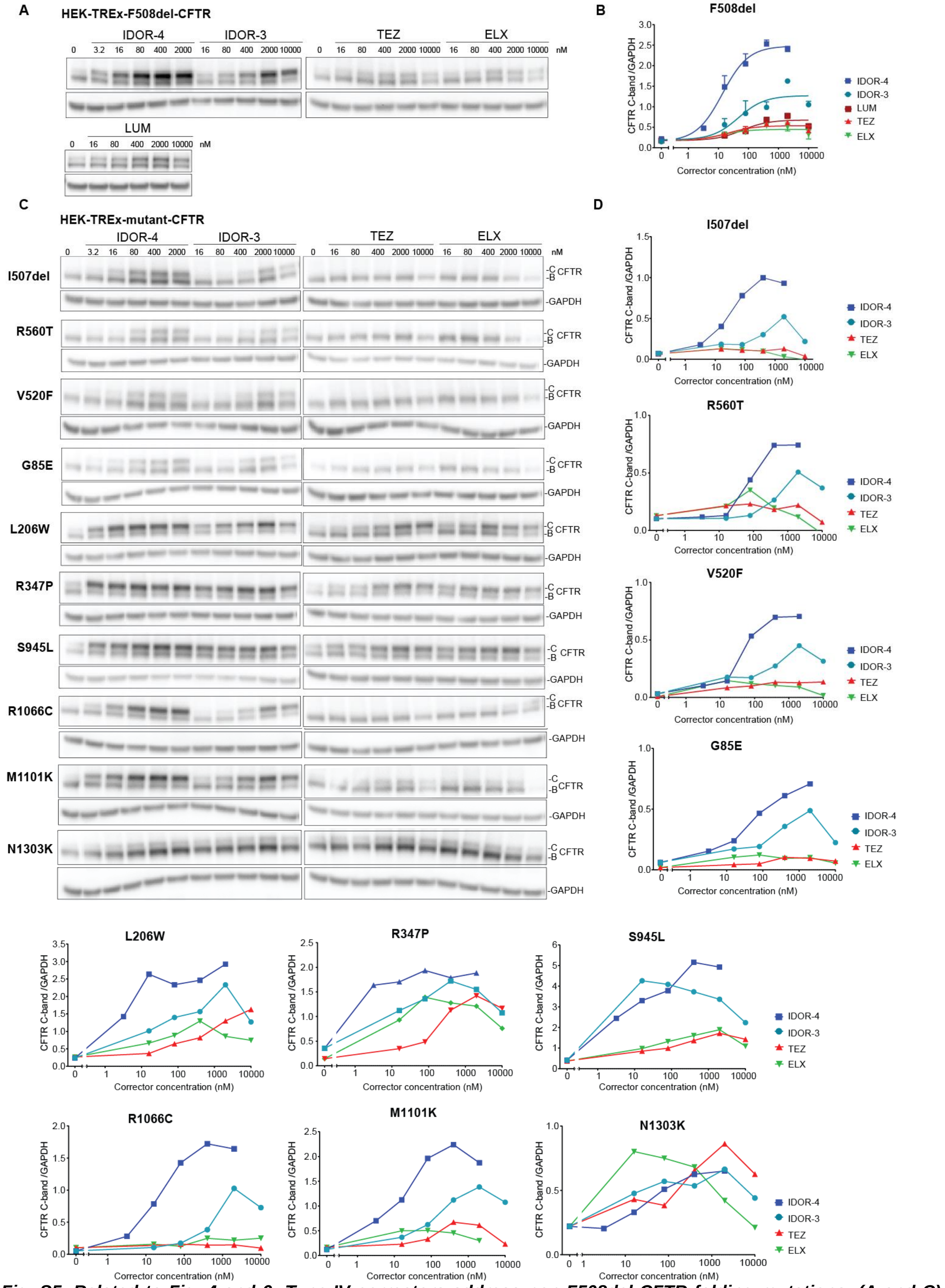

Fig. S5. Related to Fig. 4 and 6: Type-IV correctors address non-F508del CFTR folding mutations. (A and C) *Representative immuno-blot images of CFTR and GAPDH in HEK-TREx cells expressing 11 different CF-causing CFTR folding mutations and treated for 48 h with the indicated corrector concentrations (n=2 for A, n=1 for C).* ***(B and D)*** *CFTR*

C-band intensities normalized for GAPDH (n=2 for A, n=1 for C). Data in (B) are means ± SEM of the indicated number of independent experiments. In (D) values are from one experiment.

A

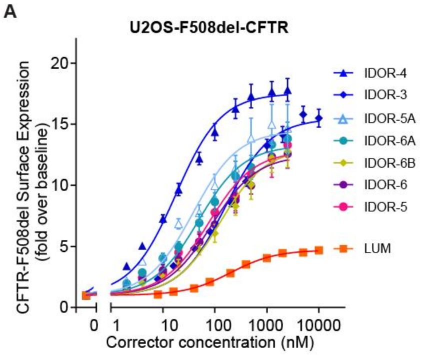

B

U2OS-F508del-CFTR

Probes		EC_{50} (nM)	E_{max} (fold baseline)
Photoactive	IDOR-5A	38	14
	IDOR-6A	49	13
	IDOR-6B	128	13
Non-photoactive	IDOR-5	75	13
	IDOR-6	86	12

C

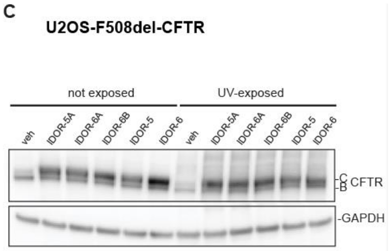

D

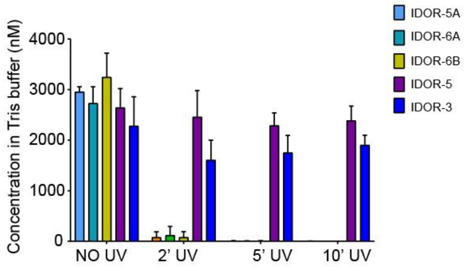

E

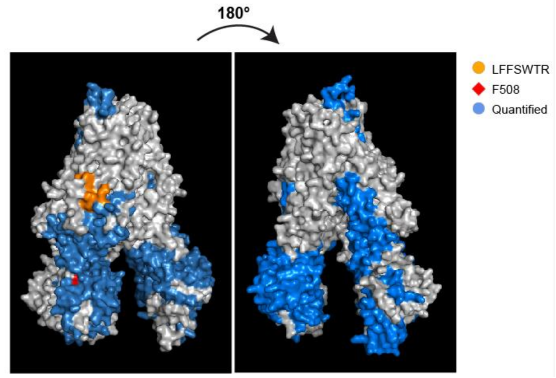

F

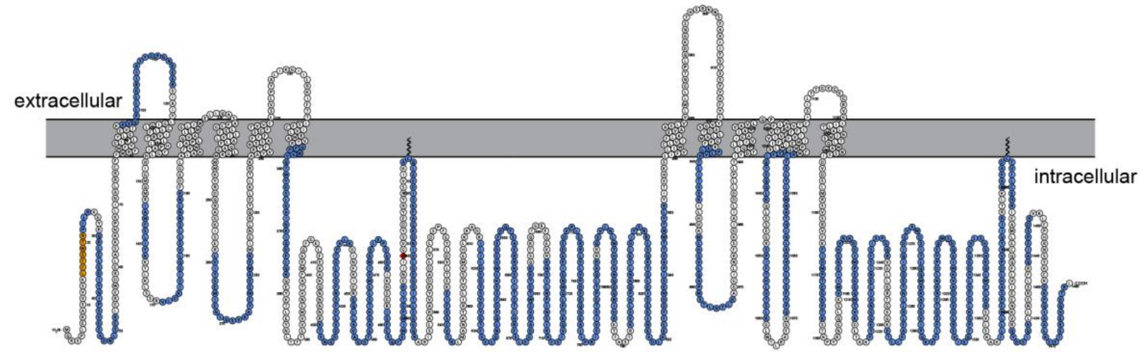

G

Rank	Peptide	group 1	group 2	p	p.adj	p.adj.signif
1	_LFFSWTRPILR_	IDOR-6 + UV	IDOR-6A + UV	1.40E-07	1.25E-05	****
2	_LFFSWTR_	IDOR-6 + UV	IDOR-6A + UV	4.41E-06	1.96E-04	***
3	_EETEEEVQDTR_	IDOR-6B NO UV	IDOR-6B + UV	0.00212	0.05778	ns
4	_LVITSEM[+16]IENIQSVK_	IDOR-6B NO UV	IDOR-6B + UV	0.00177	0.05778	ns
5	_TSLLM[+16]VIM[+16]GELEPSEGK_	IDOR-6B NO UV	IDOR-6B + UV	0.00214	0.05778	ns
6	_LFFSWTR_	IDOR-6A NO UV	IDOR-6A + UV	7.50E-04	0.061855	ns
7	_LFFSWTRPILR_	IDOR-6A NO UV	IDOR-6A + UV	0.00139	0.061855	ns
8	_QLM[+16]C[+57]LAR_	IDOR-6B NO UV	IDOR-6B + UV	0.00365	0.073913	ns
9	_TSLLM[+16]VIM[+16]GELEPSEGK_	IDOR-6A NO UV	IDOR-6A + UV	0.00318	0.09434	ns

H

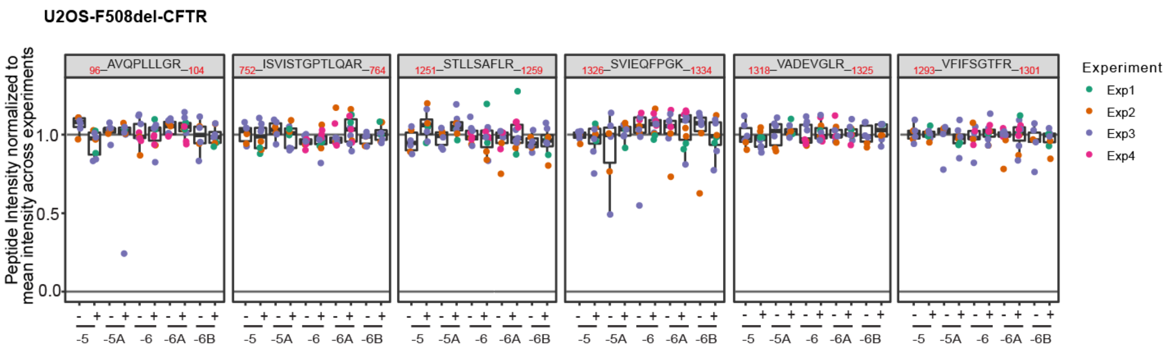

Fig. S6. Related to Fig. 5: Type-IV correctors bind to the CFTR lasso domain. (A) *F508del-CFTR cell surface expression in U2OS cells after overnight treatment with different concentrations of the indicated correctors (n=3 for each probe).* ***(B)*** *EC_{50} and E_{max} values of the three photoactivatable probes and the two parent macrocycles determined in (A).* ***(C)*** *CFTR*

and GAPDH expression levels (immuno-blotting) in U2OS F508del-CFTR cells treated for 48 h with 2 µM of the indicated probes and exposed or not to UV light for 5 min. Representative images (n=2). ***(D)*** *Concentration change of the indicated probes diluted in 50 mM Tris buffer with increasing UV exposure times (n=6).* ***(E and F)*** *3D and 2D view of the CFTR protein showing in blue all 73 peptides detected in the crosslinking approach in at least two experiments, corresponding to 53 % of the whole protein. Peptide15-21 is shown in yellow and F508 in red.* ***(G)*** *Table showing the first 9 of all identified peptides, ranked from the lowest adjusted p-value after one-sided t-test and multiple-testing correction using Benjamini Hochberg test (see Methods).* $_{15}$*LFFSWTR*$_{21}$ *and* $_{15}$*LFFSWTRPILR*$_{25}$ *were the only peptides statistically significantly reduced in any comparison and were different for samples treated with IDOR-6A versus the control probe IDOR-6 (after UV exposure). The non-adjusted p-value for these two peptides (without multiple testing correction) showed also a statistically significant difference between IDOR-6A treated samples after and without UV excitation.* ***(H)*** *Abundance of six representative CFTR peptides showing no specific effect after UV exposure or treatment with the six different probes (n=3/4). Data in (A) are mean ± SEM of the indicated number of experiments. Data in (D) are mean ± SD of 6 experimental replica. Scatter plot in (H) results from at least 3independent experiments with colored dots representing replica values. Per experiment, the intensity level of each peptide was normalized to the total CFTR signal in that sample (summed signal of all CFTR peptides).*

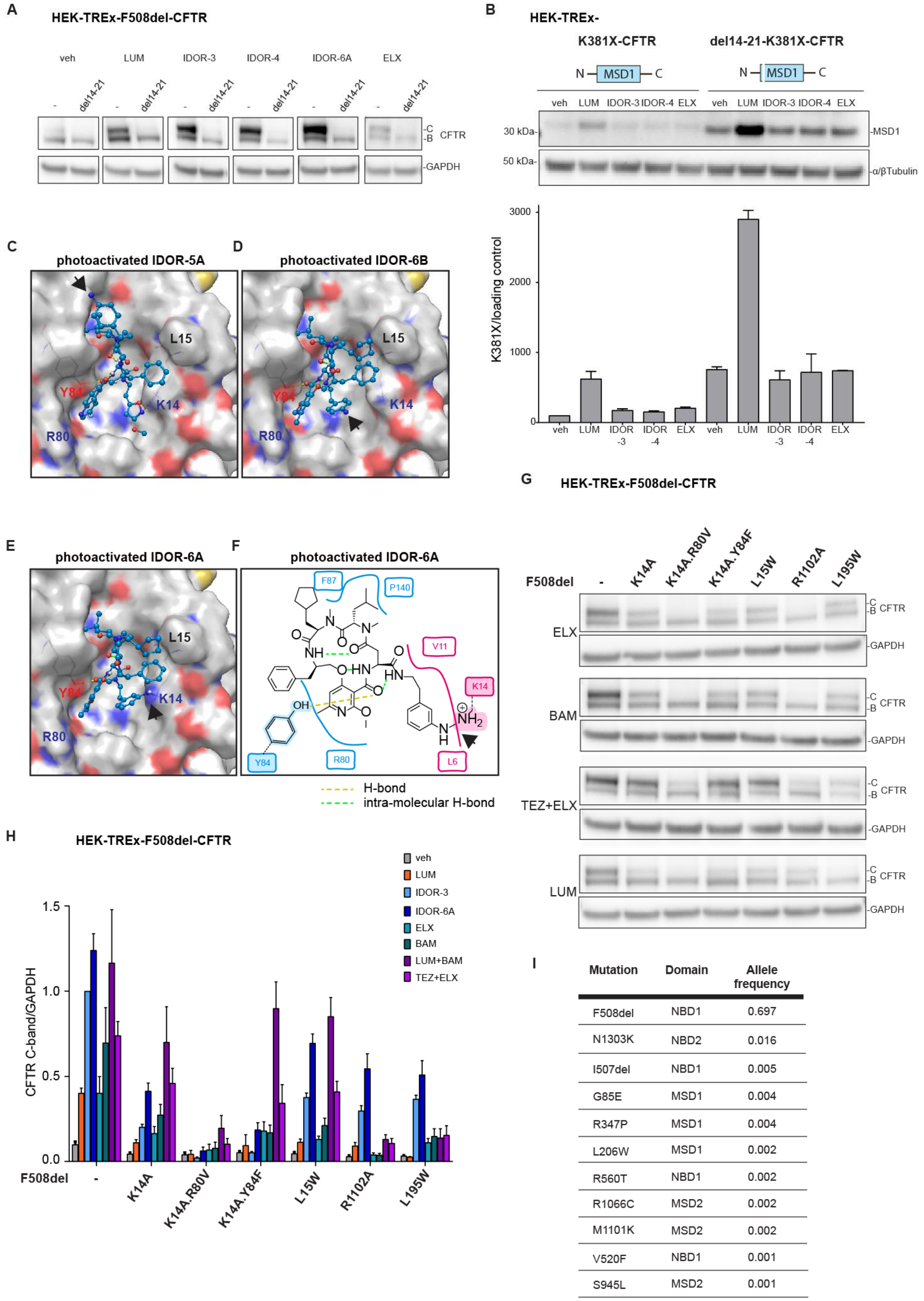

Mutation	Domain	Allele frequency
F508del	NBD1	0.697
N1303K	NBD2	0.016
I507del	NBD1	0.005
G85E	MSD1	0.004
R347P	MSD1	0.004
L206W	MSD1	0.002
R560T	NBD1	0.002
R1066C	MSD2	0.002
M1101K	MSD2	0.002
V520F	NBD1	0.001
S945L	MSD2	0.001

Fig. S7. Related to Fig. 5 / Fig. 6: Type-IV correctors bind to the CFTR lasso domain / *Type-IV correctors address non-F508del CFTR folding mutations.* **(A)** CFTR and GAPDH expression by immuno-blotting in HEK-TREx cells expressing F508del-CFTR with or without additional deletion of the amino acids 14-21 and treated for 40 h with 2 µM LUM or 2 µM IDOR-3 or 0.4 µM IDOR-4 or 2 µM IDOR-6A or 0.4 µM ELX (n=2). **(B)** Levels of truncated CFTR construct

K381X with or without amino acid 14-21 deletion expressed in HEK-TREx cells after 40 h of treatment with vehicle or 2 µM LUM or 2 µM IDOR-3 or 0.4 µM IDOR-4 or 0.4 µM ELX. Detection antibody targets MSD1. Intensities of CFTR fragment bands are shown in the graph below, normalized for the loading controls (n=2). **(C-E)** 3-dimensional views of photo-activated IDOR-5A (C), IDOR-6B (D) and IDOR-6A (E) bound to the cavity as determined by docking studies. Polar amino acid side chains are indicated in blue, red and yellow. H-bonds between the probes and CFTR are indicated as yellow dashed lines. Intramolecular H-bonds are depicted as green dashed lines. Black arrowheads point to the aryl-nitrene (activated intermediate) in each probe. **(F)** 2-dimensional view of IDOR-6A with likely cross-linking of the aryl-meta-nitrene to the K14 amine. **(G)** Site-directed mutagenesis of key amino acids in the proposed type-IV corrector binding site and effect on corrector activities: F508del-CFTR constructs containing one or two additional mutations in the site (K14A, K14A.R80V, K14A.Y84F or L15W) or the ELX binding site (R1102A) or the LUM binding site (L195W) were expressed in HEK-TREx cells then treated for 40 h with 2 µM of each indicated corrector. Representative images (at least 4 independent experiment per mutant and per corrector treatment). **(H)** Quantification of F508del-CFTR C-band intensities in (F) normalized for GAPDH (n=4-10). **(I)** Most frequent class-II folding mutations, their domain localization and allele frequency among the CF patient population. Data in (B) and (H) are mean ± SEM of the indicated number of experiments.

Table S1: Transition list for mass spectrometry-based quantification of peptides in metabolic pulse-chase and chase assays. Peptide-specific parameters for the acquisition on the Thermo Scientific Orbitrap Exactive HF-X instrument: Mass transitions, charge state (CS), polarity, retention time window, (Normalized) collision energy ((N)CE)

Mass [m/z]	CS [z]	Polarity	Start [min]	End [min]	(N)CE	(N)CE type	Comment
483.8084	2	Positive	16	17	23	NCE	AVQPLLLGR (light)
488.8125	2	Positive	16	17	23	NCE	AVQPLLLGR (heavy)
615.3461	2	Positive	14.6	15.6	21	NCE	GQLLAVAGSTGAGK (light)
619.3532	2	Positive	14.6	15.6	21	NCE	GQLLAVAGSTGAGK (heavy)
620.854	2	Positive	16.5	17.5	23	NCE	NSILNPINSIR (light)
625.8582	2	Positive	16.5	17.5	23	NCE	NSILNPINSIR (heavy)
425.2633	2	Positive	12.7	13.7	23	NCE	KFSIVQK (light)
433.2775	2	Positive	12.7	13.7	23	NCE	KFSIVQK (heavy)
862.4649	2	Positive	17.6	18.6	29	NCE	LSLVPDSEQGEAILPR (light)
867.469	2	Positive	17.6	18.6	29	NCE	LSLVPDSEQGEAILPR (heavy)
671.8881	2	Positive	15.6	16.6	23	NCE	ISVISTGPTLQAR (light)
676.8922	2	Positive	15.6	16.6	23	NCE	ISVISTGPTLQAR (heavy)
673.3687	3	Positive	16.9	17.9	23	NCE	KVSLAPQANLTELDIYSR (light)
679.3762	3	Positive	16.9	17.9	23	NCE	KVSLAPQANLTELDIYSR (heavy)
504.2978	2	Positive	18.8	19.8	19	NCE	STLLSAFLR (light)
509.302	2	Positive	18.8	19.8	19	NCE	STLLSAFLR (heavy)
537.2926	2	Positive	18	19	23	NCE	VFIFSGTFR (light)
542.2967	2	Positive	18	19	23	NCE	VFIFSGTFR (heavy)
429.7376	2	Positive	13.4	14.4	21	NCE	VADEVGLR (light)
434.7418	2	Positive	13.4	14.4	21	NCE	VADEVGLR (heavy)
446.7662	2	Positive	17.4	18.4	19	NCE	SSLLLGFR (light)
451.7703	2	Positive	17.4	18.4	19	NCE	SSLLLGFR (heavy)
452.748	2	Positive	14	15	25	NCE	IPENIYR (light)
457.7521	2	Positive	14	15	25	NCE	IPENIYR (heavy)
563.7982	2	Positive	15.3	16.3	23	NCE	VANVELYYR (light)
568.8023	2	Positive	15.3	16.3	23	NCE	VANVELYYR (heavy)
472.2534	2	Positive	14.7	15.7	23	NCE	HELIEFR (light)
477.2576	2	Positive	14.7	15.7	23	NCE	HELIEFR (heavy)
514.803	2	Positive	15.4	16.4	21	NCE	VLDAIIQEK (light)
518.8101	2	Positive	15.4	16.4	21	NCE	VLDAIIQEK (heavy)
575.3112	2	Positive	15.6	16.6	23	NCE	LLEENVSAFK (light)
579.3183	2	Positive	15.6	16.6	23	NCE	LLEENVSAFK (heavy)
569.3132	2	Positive	18.5	19.5	19	NCE	LFDLLYPEK (light)
573.3203	2	Positive	18.5	19.5	19	NCE	LFDLLYPEK (heavy)
497.2718	2	Positive	15.5	16.5	21	NCE	YNIVTLDR (light)
502.276	2	Positive	15.5	16.5	21	NCE	YNIVTLDR (heavy)
776.9298	2	Positive	19.2	20.2	27	NCE	iRT peptide 3 (light)
669.8381	2	Positive	15.2	16.2	27	NCE	iRT peptide 2 (light)
487.2567	2	Positive	11.8	12.8	27	NCE	iRT peptide 1 (light)

Table S2: Chromatographic determination of probes concentrations upon UV light exposures. Compound specific mass spectrometer parameters on the API 5500TM instrument: mass transitions, declustering potential (DP) and collision energy (CE).

Analyte	Q1 (m/z)	Q3 (m/z)	DP (V)	CE (V)
IDOR-5A	875	334	10	41
IDOR-6A	838	370	90	43
IDOR-6B	838	370	30	43
IDOR-5	834	334	10	43
IDOR-4	800	350	110	42
